# Supplementary material for: Pathogenic ACVR1R206H activation by Activin A‐induced receptor clustering and autophosphorylation
Source: EMBO J. 2021 May 18;40(14):e106317. doi: 10.15252/embj.2020106317 (PMC8280795; doi:10.15252/embj.2020106317)
Supplement: Supplementary file 1 — Appendix [file EMBJ-40-e106317-s010.pdf]

Contents for Appendix

Appendix Supplementary Methods
Sequences of all the knockout clones

Appendix Figure S1
Enzyme rates of $ACVR1^{WT}$ and $ACVR1^{R206H}$ in response to different ligands.

Appendix Figure S2
TGF-β can trigger $ACVR1^{R206H}$ signaling by an alternative mechanism.

Sequences of all knockout clones

In each case, the wild-type sequence is given on top and the mutant sequence for a particular allele is given underneath. Depending on the gene, we found between two and 4 alleles for each gene. Sequences in lower case represent mismatches to the reference sequence.

Parental ACVR1 Knockout clone 1

Allele 1

^{608}CAGTGGCTCGCCAGATTACACTGTTGGAGTGTG^{640}
CAGTGGCTCG-------------TTGGAGTGTG

Allele 2

^{608}CAGTGGCTCGCCAGATTACACTGTTGGAGTGTG^{640}
CAGTGGCTCGCCAGAT(309 nt deletion)

Parental ACVR1 knockout clone 2

Allele 1

^{608}CAGTGGCTCGCCAGATTACACTGTTGGAGTGTGT^{640}
(332 nt deletion)ACACTGTTGGAGTGTGT

Allele 2

^{608}CAGTGGCTCGCCAGATTACACTGTTGGAGTGTGT^{640}
CAGTG------------------------TGTGT

Parental ACVR2A knockout

Allele 1

^{682}TCATGGCAAAATGAATACGAAGTCTACAGTTTG^{714}
(396 nt deletion spanning Guide sequence)

Allele 2

^{682}TCATGGCAAAATGAATACGAAGTCTACAGTTTG^{714}
TCATGGCAAAATGAATACGAA(243 nt deletion)

Allele 3

^{682}TCATGGCAAAATGAATACGAAGTCTACAGTTTG^{714}
TCATGGCAAAATGAATACGAAGTCTA(18 nt deletion)

Parental ACVR2B knockout

Allele 1

^{718}AAGCACGAGAACCTGCTACAGTTCATTGCTGCCGAGAAGCGA^{759}
(29 nt deletion) ACAGTTCATTGCTGCCGAGAAGCGA

Allele 2

^{718}AAGCACGAGAACCTGCTACAGTTCATTGCTGCCGAGAAGCGA^{759}
(195 nt deletion spanning Guide sequence)

Parental ACVR2A/2B double knockout

ACVR2A

Allele 1

^{682}TCATGGCAAAATGAATACGAAGTCTACAGTTTG^{714}
(225 nt deletion) TG

Allele 2

^{682}TCATGGCAAAATGAATACGAAGTCTACAGTTT-G^{714}
TCATGGCAAAATGAATACGAAGTCTACAGTTTTG

Allele 3

^{682}TCATGGCAAAATGAATACGAAGTCTACAGTTTG^{714}
TCATGGCAAAATGAATACGAAG(23 nt deletion)

ACVR2B

Allele 1

^{718}AAGCACGAGAACCTGCTACAGTTCATTGCTGCCGAGAAGCGA^{759}
AAGCACGAGAAC(189 nt deletion)

Allele 2 not detected

HOM1 ACVR2A knockout

Allele 1

^{682}TCATGGCAAAATGAATACGAAGTCTACAGTTTG^{714}
TCATGGCAAAATGAATACGAAGTCTACAGT(270 nt deletion)

Allele 2

^{682}TCATGGCAAAATGAATACGAAGTCTACAGTTTG^{714}
TCATGGCAAAATGAA(29 nt deletion)

Allele 3

^{682}TCATGGCAAAATGAATACGAAGTCTACAGTTTG^{714}
TCATGGCAAAATGAATACGAA----------TG

HOM1 ACVR2B knockout

Allele 1

^{718}AAGCACGAGAACCTGCTACAGTTCATTGCTGCCGAGAAGCGA^{759}
AAGCcaggtggatactg(202 nt deletion)

Allele 2

^{718}AAGCACGAGAACCTGCTACAGTTCATTGCTGCCGAGAAGCGA^{759}
-------------------------TTGCTGCCGAGAAGCGA

HOM1 ACVR2A/2B double knockout

ACVR2A

Allele 1

^{682}TCATGGCAAAATGAATACGAAGTCTACAGTTTG^{714}
TCATGGCAAAATGAATACGAAGT(49 nt insertion)

Allele 2

^{682}TCATGGCAAAATGAATACGAAGTCTACAGTTTG^{714}
TCATGGCAAAATGAATACGAAGTCTACAGTTT(39 nt insertion)

Allele 3

^{682}TCATGGCAAAATGAATACGAAGTCTACAGTTTG^{714}
TCATGGCAAAATGAATACGAAGTCTACAGTT-G

ACVR2B

Allele 1

^{718}AAGCACGAGAACCTGCTACAGTTCATTGCTGCCGAGAAGCGA^{759}
AAGCACGAGAAC-------AGTTCATTGCTGCCGAGAAGCGA

Allele 2

^{718}AAGCACGAGAACCTGCTACAGTTCATTGCTGCCGAGAAGCGA^{759}

AAGCACGAG-------------------CTGCCGAGAAGCGA

Allele 3

^{718}AAGCACGAGAACCTGCTA-CAGTTCATTGCTGCCGAGAAGCGA^{759}
AAGCACGAGAACCTGCTAACAGTTCATTGCTGCCGAGAAGCGA

Parental ACVR1B/1C double knockout

ACVR1B

Allele 1

^{337}CTCAAGGAGCCTGAGCACCCGTCCATGTGGGGCCCGGTGGAGCTG^{381}
CTCAAGG-----------------------GGCCCGGTGGAGCTG

Allele 2

^{337}CTCAAGGAGCCTGAGCACCCGTCCATGTGGGGCCCGGTGGAGCTG^{381}
CTCAAGGAGCCTGAGCACC-GTCCATGTGGGGCCCGGTGGAGCTG

ACVR1C

Allele 1

^{673}TCCTCCAGAGATGAAAGATCTTGGTTTCGTGAGGCAGAAATTTAC^{717}
TCCTCCAGAGATGAAAGATCTTGGTTTC---ActttccAATTTAC

Allele 2

^{673}TCCTCCAGAGATGAAAGATCTTGGTTTCGTGAGGCAGAAATTTAC^{717}
TCCTCCAGAGATGAAAGATC--------GTGAGGCAGAAATTTAC

Allele 3

^{673}TCCTCCAGAGATGAAAGATCTTGGTTTCGTGAGGCAGAAATTTAC^{717}
TCCTCCAGAGATGAAAGATC(211 nt deletion)

HOM1 ACVR1B/1C double knockout

ACVR1B

Allele 1

^{337}CTCAAGGAGCCTGAGCACCCGTCCATGTGGGGCCCGGTGGAGCTG^{381}
CTCAAGGA-----------------TGTGGGGCCCGGTGGAGCTG

Allele 2

^{337}CTCAAGGAGCCTGAGC-ACCCGTCCATGTGGGGCCCGGTGGAGCTG^{381}

CTCAAGGAGCCTGAGCCACCCGTCCATGTGGGGCCCGGTGGAGCTG

Allele 3

^{337}CTCAAGGAGCCTGAGCACCCGTCCATGTGGGGCCCGGTGGAGCTG^{381}
CTCAAGGA-----------------TGTGGGGCCCGGTGGAGCTG

Allele 4

^{337}CTCAAGGAGCCTGAGCACCCGTCCATGTGGGGCCCGGTGGAGCTG^{381}
(38 nt deletion)TGGGGCCCGGTGGAGCTG

ACVR1C

Allele 1

^{673}TCCTCCAGAGATGAAAGATCTTGGTTTCGTGAGGCAGAAATTTAC^{717}
TCCTCCAGAGATGAAAGATCTTG------TGAGGCAGAAATTTAC

Allele 2 not detected

Parental SMAD6 knockout clone 1

Allele 1

^{961}GGGAGCACCGGACGCGCGTGGGCCGCCTCTAT-GCGGTGTACGACC^{1005}
GGGAGCACCGGACGCGCGTGGGCCGCCTCTATTGCGGTGTACGACC

Allele 2

^{961}GGGAGCACCGGACGCGCGTGGGCCGCCTCTATGCGGTGTACGACC^{1005}
GGG(227 nt insertion)

Parental SMAD6 knockout clone 2

Allele 1

^{961}GGGAGCACCGGACGCGCGTGGGCCGCCTCTATGCGGTGTACGACC^{1005}
GGGAGCACCGGACGCGCGTGGGCCGCCTCTAT(136 nt deletion)

Allele 2

^{961}GGGAGCACCGGACGCGCGTGGGCCGCCTCTATGCGGTGTACGACC^{1005}
GGGAGCACCGGACGCGCGTGGGCCGCCTCT-TGCGGTGTACGACC

Parental SMAD7 knockout clone 1

Allele 1

^{887}TTCTTCTGGAGCCTGGGGATCGGTCACACTGGTGCGTGGTGGCAT^{931}
TTCTTCTGGAGCCTGG(>300 nt insertion)

Allele 2

^{887}TTCTTCTGGAGCCTGGGGATCGGTCACACTGGTGCGTGGTGGCAT^{931}
TTCTTCTGGAGCCTGG(>300 nt deletion)

Parental SMAD7 knockout clone 2

Allele 1

^{887}TTCTTCTGGAGCCTGGGGATCGGTCACACTGGTGCGTGGTGGCAT^{931}
TTCTTCTGGAGC(109 nt insertion)

Allele 2 not detected.

Parental SMAD6/SMAD7 double knockout clone 1

SMAD7

Allele 1

^{887}TTCTTCTGGAGCCTGGGGATCGGTCACACTGGTGCGTGGTGGCAT^{931}
TTCTTCTGGAGCC----GATCGGTCACACTGGTGCGTGGTGGCAT

Allele 2

^{887}TTCTTCTGGAGCCTGGGGATCGGTCACACTGGTGCGTGGTGGCAT^{931}
TTCTTCTGGAGCCTGGG--------ACACTGGTGCGTGGTGGCAT

SMAD6

Allele 1

^{961}GGGAGCACCGGACGCGCGTGGGCCGCCTCTAT-GCGGTGTACGACC^{1005}
GGGAGCACCGGACGCGCGTGGGCCGCCTCTATTGCGGTGTACGACC

Allele 2

^{961}GGGAGCACCGGACGCGCGTGGGCCGCCTCTAT--GCGGTGTACGACC^{1005}
GGGAGCACCGGACGCGCGTGGGCCGCCTCTATTTGCGGTGTACGACC

Parental SMAD6/SMAD7 double knockout clone 2

SMAD7

Allele 1

^{887}TTCTTCTGGAGCCTGGGGATCGGTCACACTGGTGCGTGGTGGCAT^{931}
TTCTTCTGGAGCCTGG--ATCGGTCACACTGGTGCGTGGTGGCAT

Allele 2 not detected.

SMAD6

Allele 1

^{961}GGGAGCACCGGACGCGCGTGGGCCGCCTCTATGCGGTGTACGACC^{1005}
G(>300 nt deletion)

Allele 2

^{961}GGGAGCACCGGACGCGCGTGGGCCGCCTCTATGCGGTGTACGACC^{1005}
GGGAGCACCGGACGCGCGTGGGCCGCCTCTA--CGGTGTACGACC

Parental SMAD2/3 double knockout clone 1

SMAD2

Allele 1

^{799}ACTTACTCAGAACCTGCATTTT-GGTGTTCGATAGCATATTATGAATTAAAT^{849}
ACTTACTCAGAACCTGCATTTTTGGTGTTCGATAGCATATTATGAATTAAAT

Allele 2 not detected

SMAD3

Allele 1

^{703}ATCTCCTACTACGAGCTGAACCAGCGCGTCGGGGAGACATTCCACGCCTCGC^{754}
ATCTCCTACTACGAGCT------------CGGGGAGACATTCCACGCCTCGC

Allele 2

^{703}ATCTCCTACTACGAGCTGAACCAGCGCGTCGGGGAGACATTCCACGCCTCGC^{754}
ATCTCCTACTACGAG---------------------ACATTCCACGCCTCGC

Allele 3

^{703}ATCTCCTACTACGAGCTGAACCAGCGCGTCGGGGAGACATTCCACGCCTCGC^{754}
ATCTCCTACTACGAGCTGAACCAGCG--TCGGGGAGACATTCCACGCCTCGC

Parental SMAD2/3 double knockout clone 2

SMAD2

Allele 1

^{799}ACTTACTCAGAACCTGCATTTTGGTGTTCGATAGCATATTATGAATTAAAT^{849}
ACTTACTCAGAACCTGCATT--GGTGTTCGATAGCATATTATGAATTAAAT

Allele 2

^{799}ACTTACTCAGAACCTGCATTTT-GGTGTTCGATAGCATATTATGAATTAAAT^{849}
ACTTACTCAGAACCTGCATTTTTGGTGTTCGATAGCATATTATGAATTAAAT

Allele 3

^{799}ACTTACTCAGAACCTGCATTTTGGTGTTCGATAGCATATTATGAATTAAAT^{849}
ACTTACTCAGAACCTG--TTTTGGTGTTCGATAGCATATTATGAATTAAAT

SMAD3

Allele 1

^{703}ATCTCCTACTACGAGCTGAACCAGCGCGTCGGGGAGACATTCCACGCCTCGC^{754}
ATCTCCTACTACGAGCTGAACCAGCG------GGAGACATTCCACGCCTCGC

Allele 2

^{703}ATCTCCTACTACGAGCTGAACCAGCGCGTCGGGGAGACATTCCACGCCTCGC^{754}
ATCTCCTACTACGAGCTGAACCAGCGC---------------CACGCCTCGC

Allele 3

^{703}ATCTCCTACTACGAGCTGAACCAGCGCGTCGGGGAGACATTCCACGCCTCGC^{754}
ATCTCCTACTACGAGCTGAACCAGCG--TCGGGGAGACATTCCACGCCTCGC

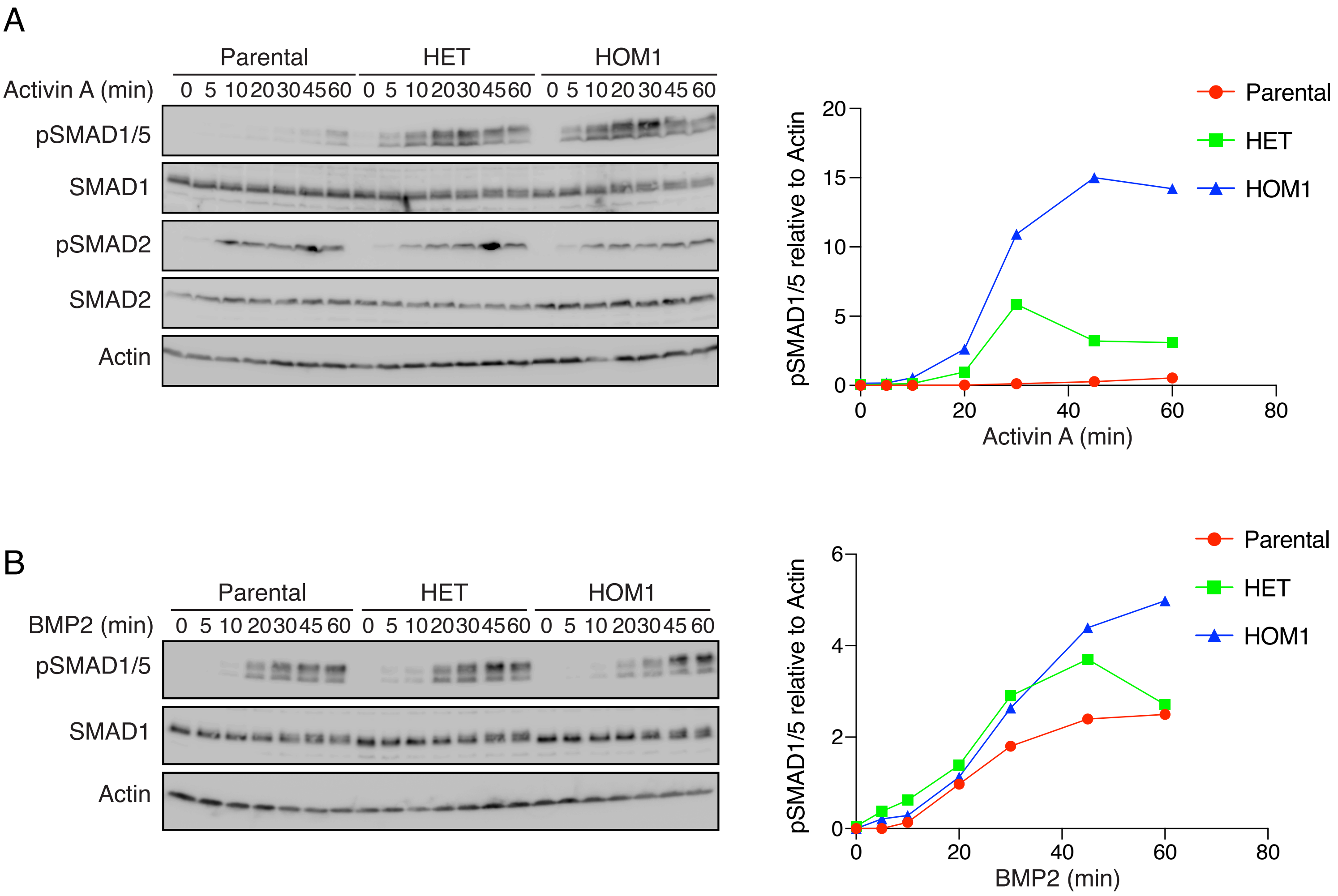

Appendix Figure S1. Enzyme rates of ACVR1^{WT} and ACVR1^{R206H} in response to different ligands.

A, B. Parental HEK293T, HET and HOM1 cells were treated with Activin A (A) or BMP2 (B) for the times indicated. In all panels, Western blots of whole cell lysates were probed with the antibodies indicated. Quantifications are the levels of pSMAD1/5 normalized to Actin and are for one representative experiment. The relative initial phosphorylation rates after Activin A induction are: $1.35 \times 10^{-5} \pm 2.13 \times 10^{-5}$, $7.94 \times 10^{-4} \pm 2.37 \times 10^{-4}$, $6.98 \times 10^{-3} \pm 2.01 \times 10^{-3}$ min^{-1} for parental, HET and HOM1 respectively (A). The relative initial phosphorylation rates after BMP2 induction are: $1.24 \times 10^{-3} \pm 1.09 \times 10^{-4}$, $1.48 \times 10^{-3} \pm 1.49 \times 10^{-4}$, $1.64 \times 10^{-3} \pm 2.98 \times 10^{-4}$ min^{-1} for parental, HET, HOM1 respectively (B).

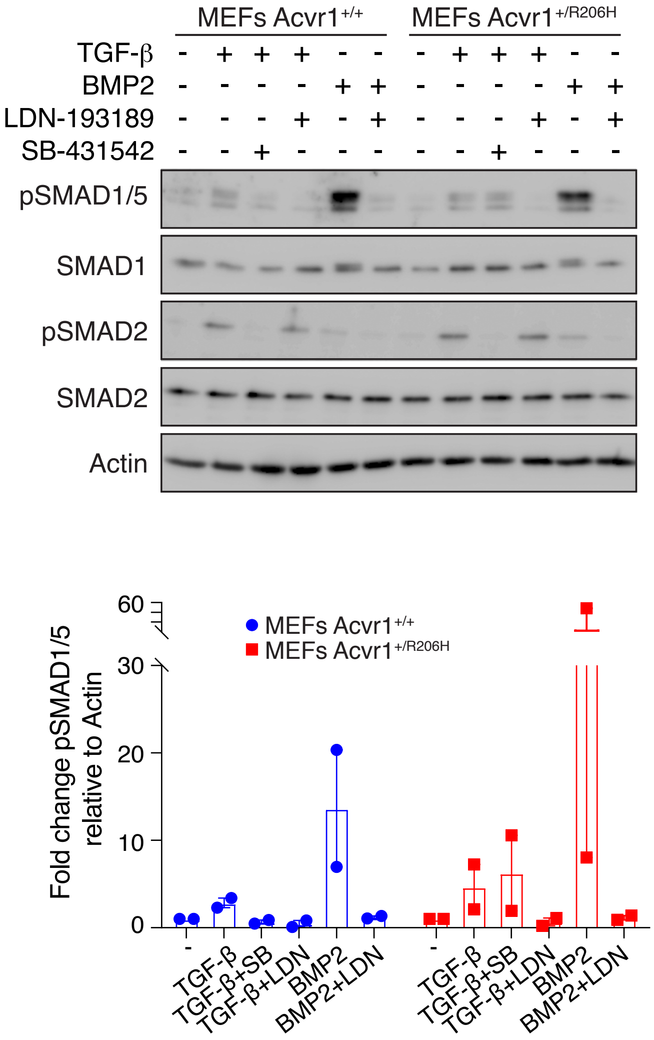

Appendix Figure S2. TGF-β can trigger ACVR1^{R206H} signaling by an alternative mechanism. MEFs from Acvr1$^{+/+}$ and Acvr1$^{+/R206H}$ mice were treated with TGF-β or BMP2 for 1 h, in the presence or absence of either 10 μM SB-431542 or 1 μM LDN-193189. In all panels, Western blots of whole cell lysates were probed with the antibodies indicated. Quantifications are the levels of pSMAD1/5 normalized to Actin, expressed as fold change relative to untreated of each cell line, and are the average ± SEM of 2 independent experiments.
