# Supplementary material for: Pathogenic ACVR1R206H activation by Activin A‐induced receptor clustering and autophosphorylation
Source: EMBO J. 2021 May 18;40(14):e106317. doi: 10.15252/embj.2020106317 (PMC8280795; doi:10.15252/embj.2020106317)
Supplement: Supplementary file 2 — Expanded View Figures PDF [file EMBJ-40-e106317-s019.pdf]

Expanded View Figures

Figure EV1. Knock-in of ACVR1^{R206H} and its responsiveness to TGF-β family ligands.

A, B Sanger sequencing traces of the ACVR1 locus at nucleotides 604-630 in HET (A) or HOM1 (B). In each case, the wild-type sequence is shown for reference.

C Parental HEK293T, HOM2, and KO2 cells were treated with the indicated ligands for 1 h. Fold changes indicated are the levels of pSMAD1/5 relative to actin, expressed as fold change relative to the untreated parental HEK293T sample. A representative experiment is shown. In all panels, Western blots of whole cell lysates were probed with the antibodies indicated. Act, Activin and KO2, ACVR1 knockout clone 2.

Source data are available online for this figure.

© 2021 The Authors

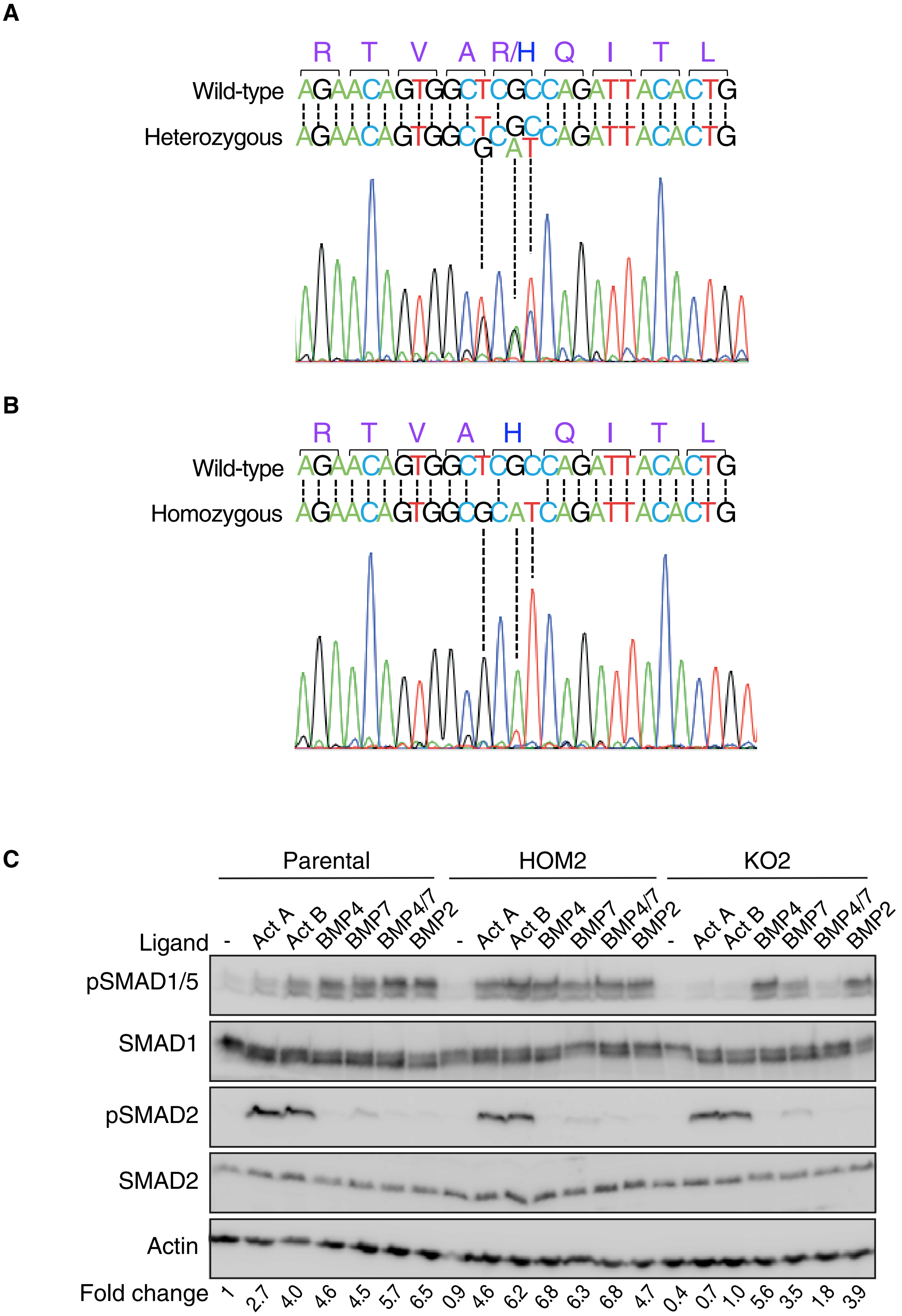

Figure EV1.

 © 2021 The Authors

Figure EV2. Kinetics of SMAD1/5 phosphorylation.

A Parental HEK293T and HOM2 cells were treated with Activin A or Activin B for the indicated times. Quantifications are presented on the right. These are the same quantifications as shown in Fig 2A, but here they are shown expressed as fold changes relative to 1 h of ligand stimulation to see more clearly how signaling attenuated over time.

B Parental HEK293T, HET, and HOM1 cells were treated with Activin A for the indicated times, and gene expression was measured by qPCR. Expression of *ID1*, *ID3*, and *ATOH8* was normalized to *GAPDH* and is presented as fold change relative to parental untreated. Data are the average ± SEM of 4–7 experiments.

C Parental HEK293T and HOM1 cells were treated with Activin A for the indicated times, up to 24 h. Quantifications are presented on the right and are the averaged normalized intensities ± SEM of 3 independent experiments. Note that the 0-h, 1-h, 4-h, and 8-h data from these 3 experiments are also included in the data shown in (A).

D Parental HEK293T and S2/3 dKO2 cells were treated with Activin A for the indicated times.

E Parental HEK293T cells were treated with Activin A for the indicated times, and gene expression was measured by qPCR. Expression of *SMAD6* and *SMAD7* was normalized to *GAPDH* and is presented as fold change relative to untreated. Data are the average ± SEM of 4 experiments.

F Parental HEK293T, S6 KO1, S6 KO2, S7 KO1, and S7 KO2 cells were treated with Activin A for the indicated times.

Data information: In (A, C, D, and F), Western blots of whole cell lysates were probed with the antibodies indicated. Quantifications are the average normalized intensities ± SEM of 3 independent experiments. In all cases, quantifications are the levels of pSMAD1/5 normalized to actin, additionally expressed as fold changes relative to 1 h of ligand stimulation. The *P*-values are from two-way ANOVA with Dunnett's *post hoc* test. ns, not significant; *$P < 0.05$; **$P < 0.01$; ***$P < 0.001$; and ****$P < 0.0001$. In (B), the *P*-values are from one-way ANOVA with Dunnett's *post hoc* test. ns, not significant; *$P < 0.05$; **$P < 0.01$; ***$P < 0.001$; and ****$P < 0.0001$. Act, Activin; S2/3, SMAD2/3; S6, SMAD6; S7, SMAD7; KO, knockout; and dKO, double knockout.

Source data are available online for this figure.

© 2021 The Authors

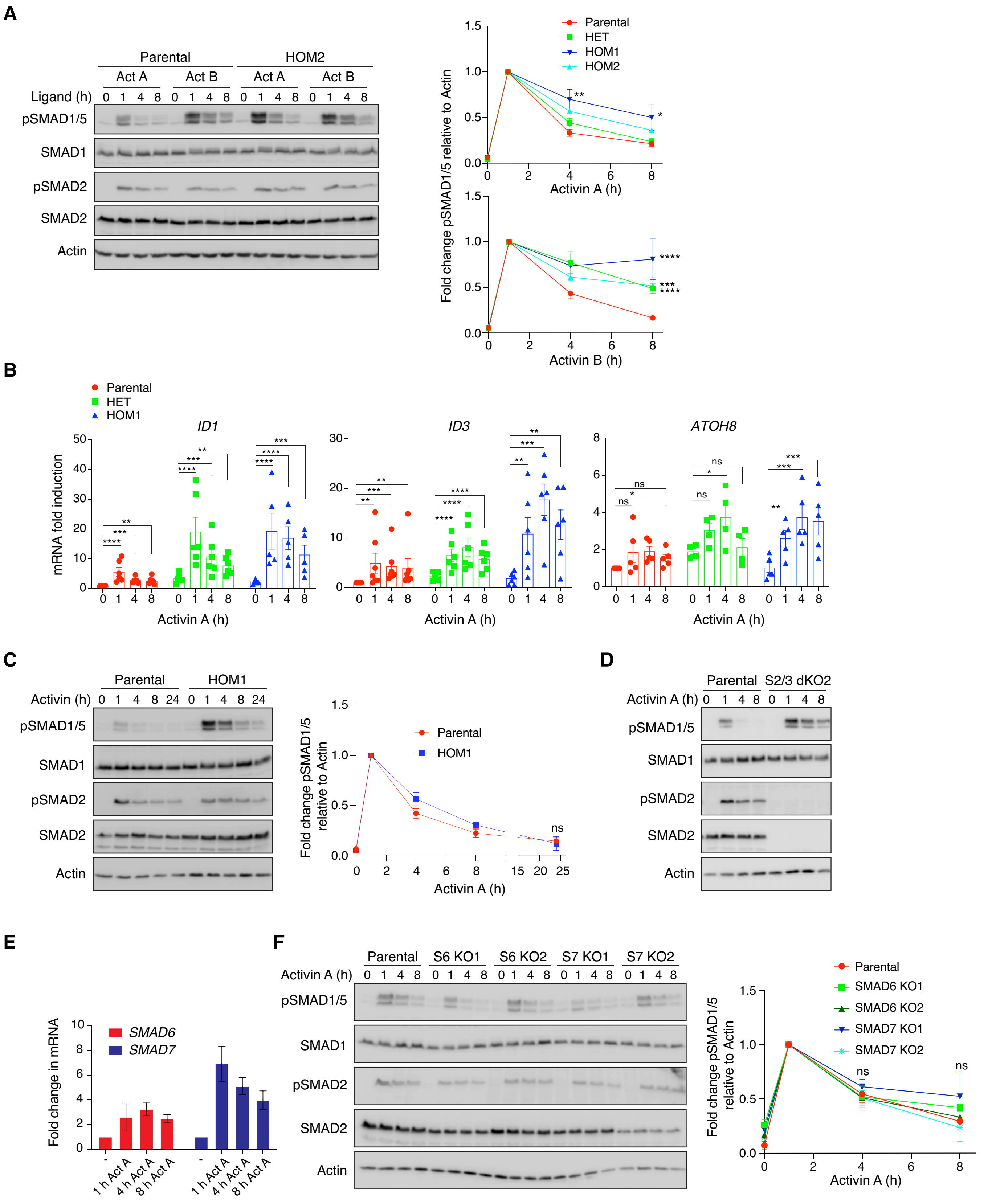

Figure EV2.

 © 2021 The Authors

Figure EV3. ACVR1 signaling from endosomes and recycling to the cell surface.

A Parental HEK293T, HET, and HOM1 cells were untreated (a), stimulated with Activin A for 1 h (b), and then left (upper panel) or chased with follistatin (lower panel) for a further 0.5 h (c), 1 h (d), 1.5 h (e), 2 h (f), or 3 h (g) as indicated in the experimental scheme. Quantifications are the levels of pSMAD1/5 normalized to actin levels expressed as fold changes relative to the level after 1 h of Activin treatment for one representative experiment.

B Parental HEK293T and HOM1 cells were untreated (a), treated with Activin A for 2 h (b), and then left untreated (d) or treated with follistatin (c) for a further 2 h. Subsequently, follistatin was washed out and the cells re-stimulated with Activin A for a further 1 h (e). As controls, cells were simultaneously treated with Activin A and follistatin (f) to confirm the efficacy of follistatin or pre-treated with follistatin for 2 h followed by follistatin wash out and re-stimulation with Activin A for 1 h (g) to assay for efficient wash out.

C Parental HEK293T and HOM1 cells were untreated or treated with Activin A for 1 or 8 h. After 8 h, cells were re-stimulated with Activin A, BMP2, or BMP4/7 for a further 1 h. Naive cells were also treated with BMP2 and BMP4/7 for 1 h as controls. Quantifications are the levels of pSMAD1/5 normalized to actin, expressed as fold change relative to 1-h BMP2 stimulation of the parental HEK293T cells, and are the average ± SEM of 3 independent experiments.

Data information: In all panels, Western blots of whole cell lysates were probed with the antibodies indicated. The *P*-values in (C) are from one-way ANOVA with Sidak's *post hoc* test. ns, not significant; $^{**}P < 0.01$; and $^{***}P < 0.001$.

Source data are available online for this figure.

© 2021 The Authors

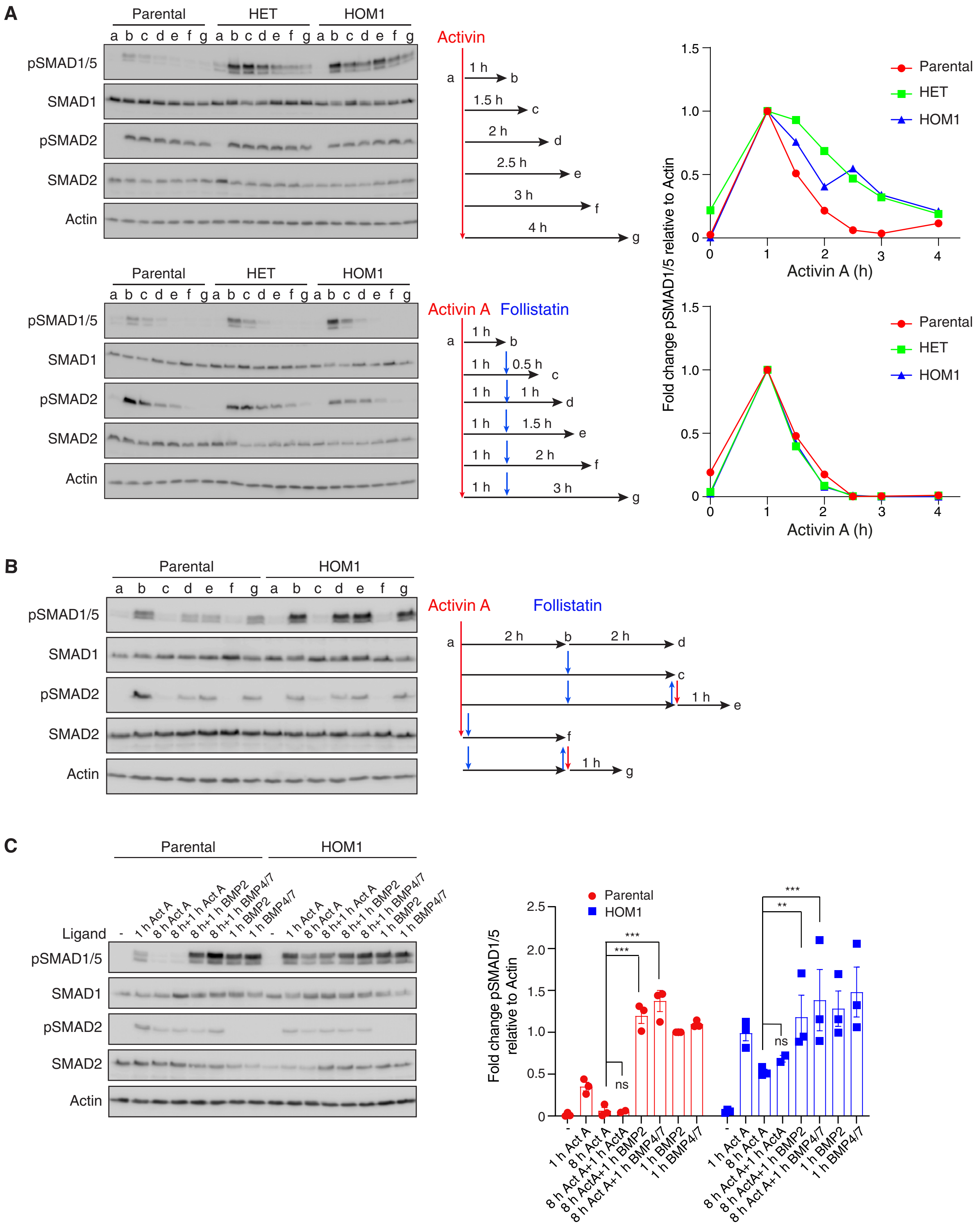

Figure EV3.

 © 2021 The Authors

Figure EV4. Neutralization of Activin A signaling and Activin A clustering in DIPG cells.

A Parental HEK293T and HOM1 cells were treated with Activin A either with or without pre-incubation with both 0.7 μg/ml of rhACVR2A Fc and 9 μg/ml of rhACVR2B Fc for 15 min. ACVR2A/B dKO and HOM1 ACVR2A/B dKO cells were also treated with Activin A for 1 h. Fold changes indicated are the levels of pSMAD1/5 (upper panel) or pSMAD2 (lower panel) relative to actin, expressed as fold change relative to the untreated parental HEK293T sample. A representative experiment is shown. In all panels, Western blots of whole cell lysates were probed with the antibodies indicated.

B–F His-Activin A-Atto647N was imaged free in solution with no cells (B) or with ICR-B169 cells (C), ICR-B169 cells pre-incubated with 1.5 μg/ml of rhACVR2A Fc and 15 μg/ml of rhACVR2B Fc (D), HSJD-DIPG-007 cells (E), DIPG-007 cells pre-incubated with 1.5 μg/ml of rhACVR2A Fc and 15 μg/ml of rhACVR2B Fc (F). Number and Brightness (N&B) analysis was performed on raster-scanned images, and a representative experiment is shown. Panel i, Brightness (B) *vs* intensity 2D plots. Red rectangular box, pixels that contribute to brightness of free, dimeric His-Activin A-Atto647N in solution. Green rectangular box, pixels that contribute to His-Activin A-Atto647N clustered into putative tetramers. Blue and pink rectangular boxes, pixels that contribute to His-Activin A-Atto647N in higher-order clusters. Panel ii, Selection map of the image captured reconstructed with the pixels in red, green, blue, and pink rectangular boxes shown in the upper panel (i). Panel iii, B histograms, frequency distribution of the number of pixels across various brightness of His-Activin A-Atto647N of the selected image. Bav indicates the average brightness for each image. Panel iv, cumulative B histograms from a representative experiment with 4–5 independent regions of interest.

Source data are available online for this figure.

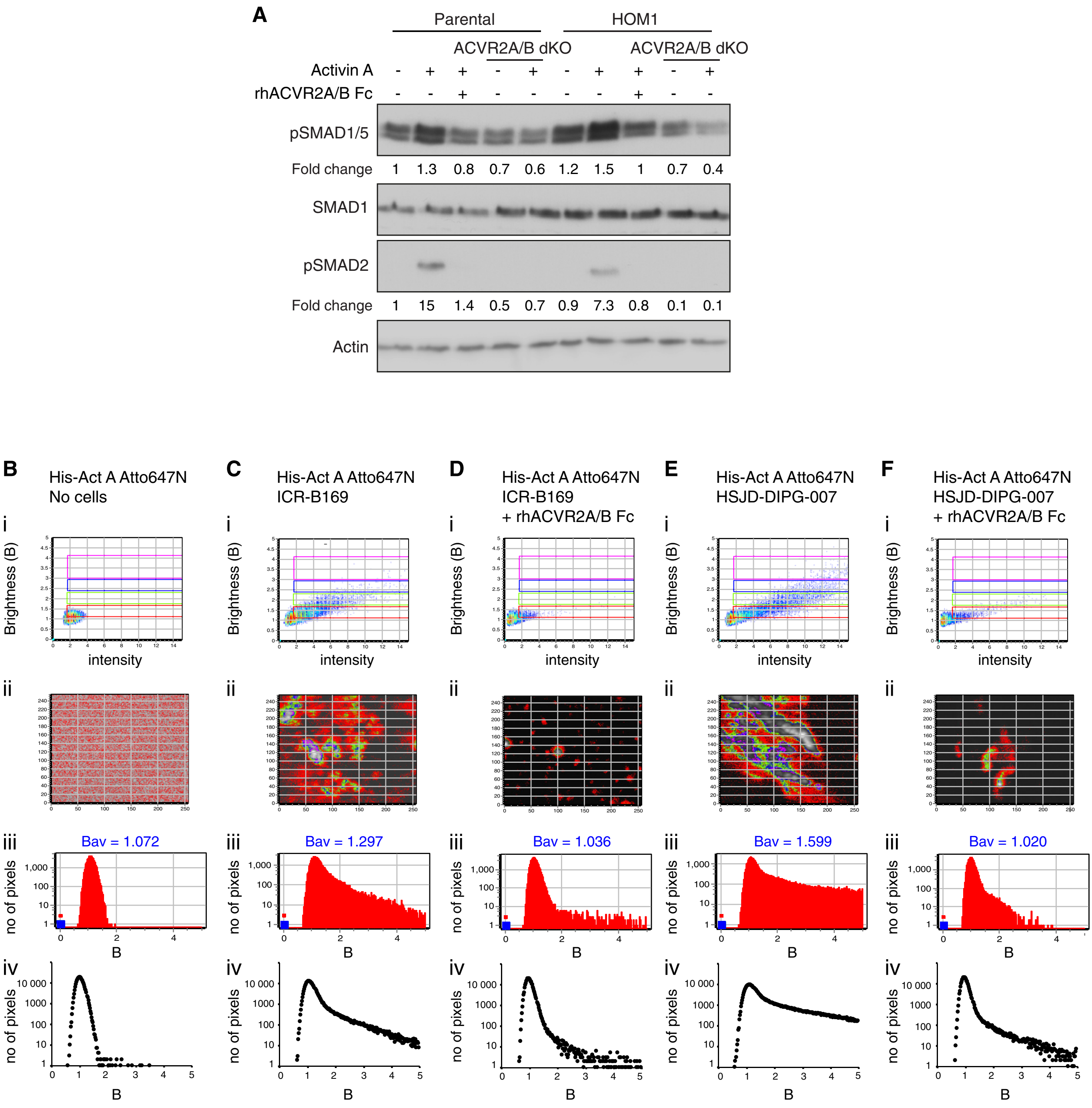

Figure EV4.

 © 2021 The Authors

Figure EV5. TIRF imaging of Activin A-induced clustering on artificial lipid bilayers.

A Mobile His-Activin A-CF640R on a planar lipid bilayer was imaged for 7.5 min; 3 time points are shown. Scale bar 10 μm.
B HEK293T HOM1 cells were plated on a planar lipid bilayer containing His-Activin A-CF640R and an automated time-lapse was started, where 3 positions within a well were imaged during a 7.5-min time period; 3 time points for 1 position are shown. TIRF, upper panels and brightfield, lower panels. Scale bar 10 μm.
C The cluster intensity from all positions was quantified at 5 time points indicated and plotted as individual cluster values. Cells + bilayer denotes bilayer areas under the cells, as identified from the brightfield images, and bilayer denotes areas where no cells are present. The horizontal gray bar denotes the mean.
D Mean cluster intensity per cell over time. Quantifications are the mean cluster intensities at 5 time points of 13 cells (NIP + cells), 61 cells (Activin A + cells), and 16 bilayer areas ± SEM, from a representative experiment imaged as in (A). The *P*-values are from *F*-test. ns, not significant and ***$P < 0.001$.

Source data are available online for this figure.

© 2021 The Authors

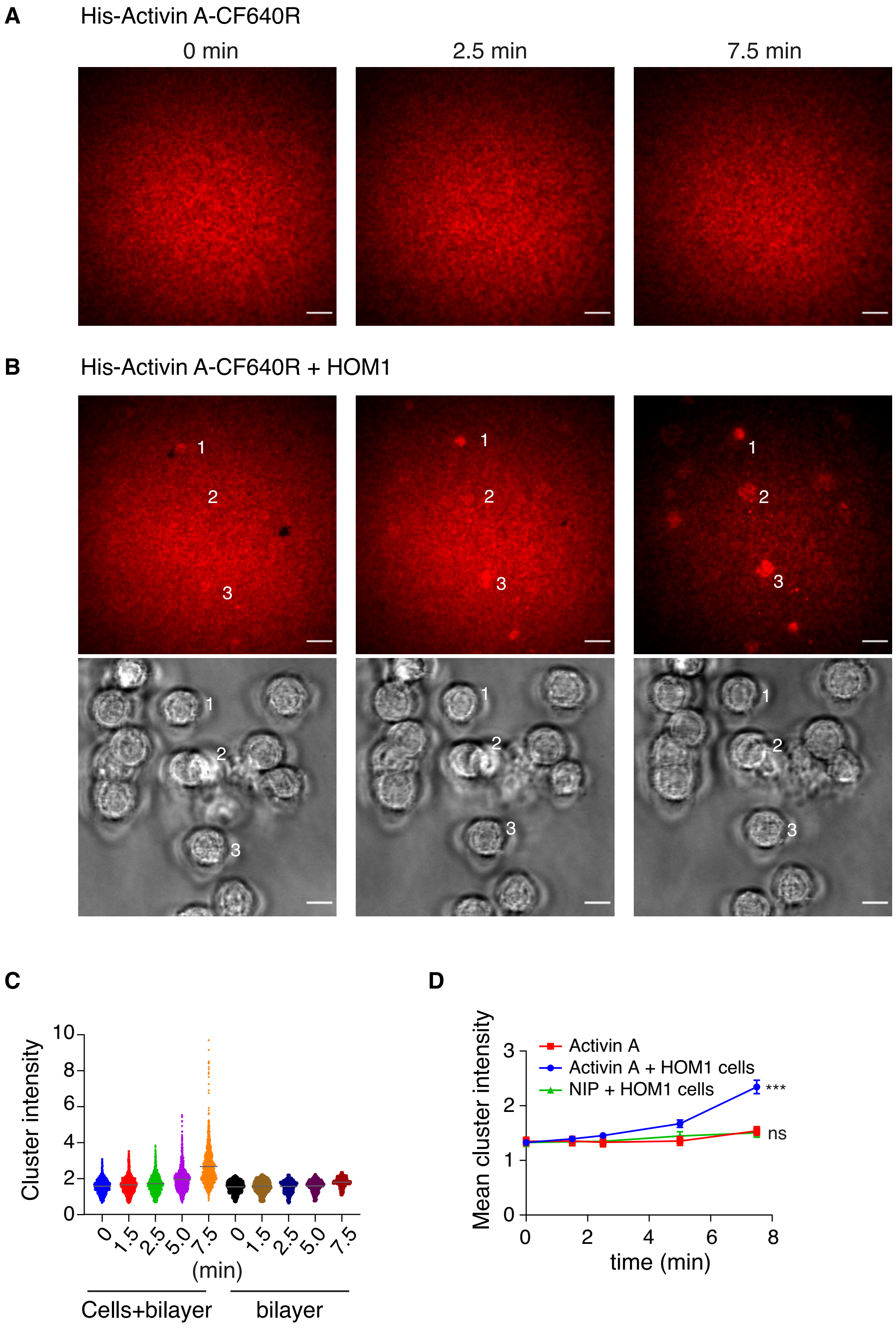

Figure EV5.

© 2021 The Authors
