# Supplementary material for: Pathogenic ACVR1R206H activation by Activin A‐induced receptor clustering and autophosphorylation
Source: EMBO J. 2021 May 18;40(14):e106317. doi: 10.15252/embj.2020106317 (PMC8280795; doi:10.15252/embj.2020106317)
Supplement: Supplementary file 3 — Table EV1 [file EMBJ-40-e106317-s002.docx]

**Table EV1 – Oligonucleotides**

| **Gene target** | **Accession No.** | **Purpose** | **Target sequence location (coding sequence)** | **Oligonucleotide** |
| --- | --- | --- | --- | --- |
| *ACVR1* | NM_001105.4 | Homology directed repair gRNA, forward oligonucleotide | 621–640 | CACCGCACACTCCAACAGTGTAATC |
|  |  | Homology directed repair gRNA, reverse oligonucleotide | 621–640 | AAACGATTACACTGTTGGAGTGTGC |
| *ACVR1B* | NM_020328.3 | CRISPR/Cas9 mediated knockout, forward oligonucleotide | 349-368 | CACCGCCCCACATGGACGGGTGCTC |
|  |  | CRISPR/Cas9 mediated knockout, reverse oligonucleotide | 349-368 | AAACGAGCACCCGTCCATGTGGGGC |
| *ACVR1C* | NM_145259.3 | CRISPR/Cas9 mediated knockout, forward oligonucleotide | 684-703 | CACCGTGAAAGATCTTGGTTTCGTG |
|  |  | CRISPR/Cas9 mediated knockout, reverse oligonucleotide | 684-703 | AAACCACGAAACCAAGATCTTTCAC |
| *ACVR2A* | NM_001278579.1 | CRISPR/Cas9 mediated knockout, forward oligonucleotide | 697–716 | CACCGTACGAAGTCTACAGTTTGCC |
|  |  | CRISPR/Cas9 mediated knockout, reverse oligonucleotide | 697–716 | AAACGGCAAACTGTAGACTTCGTAC |
| *ACVR2B* | NM_001106.4 | CRISPR/Cas9 mediated knockout, forward oligonucleotide | 732–751 | CACCGCGGCAGCAATGAACTGTAGC |
|  |  | CRISPR/Cas9 mediated knockout, reverse oligonucleotide | 732–751 | AAACGCTACAGTTCATTGCTGCCGC |
| *SMAD2* | NM_005901.6 | CRISPR/Cas9 mediated knockout, forward oligonucleotide | 814–833 | CACCGCTATCGAACACCAAAATGC |
|  |  | CRISPR/Cas9 mediated knockout, reverse oligonucleotide | 814–833 | AAACGCATTTTGGTGTTCGATAGC |
| *SMAD3* | NM_005902.4 | CRISPR/Cas9 mediated knockout, forward oligonucleotide | 726–745 | CACCGGAATGTCTCCCCGACGCGC |
|  |  | CRISPR/Cas9 mediated knockout, reverse oligonucleotide | 726–745 | AAACGCGCGTCGGGGAGACATTCC |
| *SMAD6* | NM_005585.5 | CRISPR/Cas9 mediated knockout, forward oligonucleotide | 1038–1057 | CACCGCCTGGTCGTACACCGCATAG |
|  |  | CRISPR/Cas9 mediated knockout, reverse oligonucleotide | 1038–1057 | AAACCTATGCGGTGTACGACCAGGC |
| *SMAD7* | NM_005904.3 | CRISPR/Cas9 mediated knockout, forward oligonucleotide | 766–785 | CACCGCACCAGTGTGACCGATCCCC |
|  |  | CRISPR/Cas9 mediated knockout, reverse oligonucleotide | 766–785 | AAACGGGGATCGGTCACACTGGTGC |
| *ACVR1* | - | Repair template | - | T*T*GGATCATTCGTGTACATCAGGAAGTGGCTCTGGTCTTCCTTTTCTGGTACAAAGAACAGTGGCGCATCAGATTACACTGTTGGAGTGTGTCGGTAA*T*T |
| *ACVR1* | Genomic | Universal Reverse Primer_Clone Screening | - | GAAGTTCATTGTCCAAAAGCCTA |
| *ACVR1* | Genomic | ACVR1^WT^ Forward Primer_Clone Screening | - | GTACAAAGAACAGTGGCTCGC |
| *ACVR1* | Genomic | ACVR1^R206H^ Forward Primer_Clone screening | - | GTACAAAGAACAGTGGCGCAT |
| *ACVR2A* | NM_001278579.1 | Forward primer for cloning of full length ACVR2A for expression, encodes a NheI site | 1–21 | GCTAGCATGGGAGCTGCTGCAAAGTTG |
| *ACVR2A* | NM_001278579.1 | Reverse primer for cloning of full length ACVR2A for expression, encodes a short GS linker, FLAG tag, stop codon and HindIII site | 1525–1539 | AAGCTTTCACTTGTCGTCATCGTCTTTGTAGTCGCTACTACCTAGACTAGATTCTTT |
| *ACVR2A* | NM_001278579.1 | Site directed mutagenesis of the K219R mutation, forward primer | 646–666 | GTGGCTGTCCGAATATTTCCA |
|  |  | Site directed mutagenesis of the K219R mutation, reverse primer | 646–666 | TGGAAATATTCGGACAGCCAC |
| *ACVR1* | NM_001105.4 | Site directed mutagenesis of the R206H mutation, forward primer | 601–634 | CAAAGAACAGTGGCTCACCAGATTACACTGTTGG |
|  |  | Site directed mutagenesis of the R206H mutation, reverse primer | 601–634 | CCAACAGTGTAATCTGGTGAGCCACTGTTCTTTG |
| *GAPDH* | NM_002046.7 | qPCR, forward primer | 855–874 | CTTCAACAGCGACACCCACT |
|  |  | qPCR, reverse primer | 1003-1022 | GTGGTCCAGGGGTCTTACTC |
| *SMAD6* | NM_005585.5 | qPCR, forward primer | 780–799 | CTGCAACCCCTACCACTTCA |
|  |  | qPCR, reverse primer | 945–964 | ACATGCTGGCGTCTGAGAAT |
| *SMAD7* | NM_005904.3 | qPCR, forward primer | 592–611 | CTTAGCCGACTCTGCGAACT |
|  |  | qPCR, reverse primer | 748–767 | CCAGGCTCCAGAAGAAGTTG |
| *ID1* | NM_002165.4 | qPCR, forward primer | 421-439 | GCCGAGGCGGCATGCGTTC |
|  |  | qPCR, reverse primer | 496-515 | CTTGCCCCCTGGATGGCTGG |
| *ID3* | NM_002167.5 | qPCR, forward primer | 280-298 | GGCCCCCACCTTCCCATCC |
|  |  | qPCR, reverse primer | 380-400 | GCCAGCACCTGCGTTCTGGAG |
| *ATOH8* | NM_032827.7 | qPCR, forward primer | 813-832 | CCTGAGGATCGCCTGTAACT |
|  |  | qPCR, reverse primer | 862-881 | TGGTCGGCACTGTAGTCAAG |
| *Opto-ACVR1B** | - | Opto-ACVR1B* forward cloning primer for myristoylation domain with 5' BamHI site | - | GGCGCCGGATCCATGGGGAGTAGCAAG |
| *Opto-ACVR1B** | - | Reverse primer encodes linker and N-terminus of activated ACVR1B kinase domain | - | GACACGCTGATGATAGTTTCCGCCACCACTTCC |
| *Opto-ACVR1B** | - | Forward primer encodes linker and N-terminus of activated ACVR1B kinase domain | - | GGAAGTGGTGGCGGAAACTATCATCAGCGTGTC |
| *Opto-ACVR1B** | - | Reverse primer encodes linker and C-terminus of activated ACVR1B kinase domain | - | AGAACCTCCTCCACCGATCTTCACGTCTTCCTG |
| *Opto-ACVR1B** | - | Forward primer encodes linker and C-terminus of activated ACVR1B kinase domain | - | CAGGAAGACGTGAAGATCGGTGGAGGAGGTTCT |
| *Opto-ACVR1B** | - | Opto-ACVR1B* reverse primer with HA tag, stop codon and XhoI site | - | CTCGAGTTATGCGTAGTCTGGTACG |
