# Supplementary material for: Pathogenic ACVR1R206H activation by Activin A‐induced receptor clustering and autophosphorylation
Source: EMBO J. 2021 May 18;40(14):e106317. doi: 10.15252/embj.2020106317 (PMC8280795; doi:10.15252/embj.2020106317)
Supplement: Supplementary file 4 — Table EV2 [file EMBJ-40-e106317-s015.docx]

**Table EV2 - Reagents and Tools**

| **Reagent/Resource** | **Reference or Source** | **Identifier or Catalog Number** |
| --- | --- | --- |
| **Experimental Models**  *Cell lines* |  |  |
| HEK293T | Francis Crick Institute Cell Services | N/A |
| HET | This study | N/A |
| HOM1 | This study | N/A |
| HOM2 | This study | N/A |
| ACVR1 KO1 | This study | N/A |
| ACVR1 KO2 | This study | N/A |
| S2/3 dKO1 | This study | N/A |
| S2/3 dKO2 | This study | N/A |
| S6 KO1 | This study | N/A |
| S6 KO2 | This study | N/A |
| S7 KO1 | This study | N/A |
| S7 KO2 | This study | N/A |
| S6/7 dKO1 | This study | N/A |
| S6/7 dKO2 | This study | N/A |
| ACVR1B/C dKO | This study | N/A |
| HOM1 ACVR1B/C dKO | This study | N/A |
| ACVR2A KO | This study | N/A |
| ACVR2B KO | This study | N/A |
| ACVR2A/B dKO | This study | N/A |
| HOM1 ACVR2A KO | This study | N/A |
| HOM1 ACVR2B KO | This study | N/A |
| HOM1 ACVR2A/B dKO | This study | N/A |
| NIH-3T3 | Richard Treisman, Francis Crick Institute, London | N/A |
| MEFs - Acvr1^+/+^ | Eileen M. Shore  University of Pennsylvania | Culbert et al, 2014. |
| MEFs - Acvr1^+/R206H^ | Eileen M. Shore  University of Pennsylvania | Culbert et al, 2014. |
| HSJD-DIPG-007 | Chris Jones, ICR, London | Taylor et al., 2014 |
| ICR-B169 | Chris Jones, ICR, London | Izquierdo et al., 2021 |
| **Recombinant DNA** |  |  |
| pcDNA3.1 Hygro (+) | Thermo Fisher | V87020 |
| pcDNA3.1 Hygro (+) - ACVR2A^WT^ (full length human ACVR2A^WT^) | This study | N/A |
| pcDNA3.1 Hygro (+) - ACVR2A^KR^ (full length human ACVR2A^KR^) | This study | N/A |
| pCS2+ | Richard Harland, Berkeley | N/A |
| PX458 | Ran et al., 2013 | Addgene plasmid #48138 |
| Opto-ACVR1^WT^ (human ACVR1^WT^ kinase domain) | Ramachandran et al., 2018 | N/A |
| Opto-ACVR1^R206H^ (human ACVR1^R206H^ kinase domain) | This study | N/A |
| Opto-ACVR1B* (mouse ACVR1B* kinase domain) | This study | N/A |
| FLAG-SMAD1 (human) | Lechleider et al., 2001 | N/A |
| GFP-SMAD3 (human) | Nicolas et al., 2004 | N/A |
| **Antibodies** |  |  |
| phospho-SMAD1/5 (Rabbit polyclonal, Western, 1:500-1:1000) | Cell Signaling Technologies | Cat#9511; RRID: AB_331671 |
| phospho-SMAD2 (Rabbit monoclonal, Western, 1:500) | Cell Signaling Technologies | Cat#3108; RRID: AB_490941 |
| phospho-SMAD3 (Rabbit monoclonal, Western, 1:1000) | Abcam | Cat#ab52903; RRID:AB_882596 |
| SMAD3 (Rabbit polyclonal, Western, 1:1000) | Abcam | Cat#ab28379; RRID:AB_2192903 |
| SMAD2/3 (Mouse monoclonal, Western, 1:1000) | BD Biosciences | Cat#610842; RRID: AB_398161 |
| SMAD1 (Rabbit polyclonal, Western, 1:1000) | Thermo Fisher | Cat#385400; RRID:AB_2533373 |
| Actin (Mouse monoclonal, Western, 1:10000) | Sigma | Cat#3853; RRID:AB_262137 |
| TUBULIN (Rat monoclonal, Western, 1:5000) | Abcam | Cat#ab6160; RRID:AB_305328 |
| HA tag (Rat monoclonal, Western, 1:500) | Roche | Cat#11802600; RRID:AB_2314622 |
| FLAG tag (Rat monoclonal, Western, 1:1000) | Thermo Scienctific | Cat#MA1-142; RRID:AB_2536846 |
| Goat anti-rabbit HRP  (Western, 1:3000) | Dako | Cat#P0448; RRID: AB_2617138 |
| Goat anti-mouse HRP  (Western, 1:3000-1:10000) | Dako | Cat#P0447; RRID:AB_2617137 |
| Donkey anti-rat HRP  (Western, 1:3000) | Jackson Lab | Cat#712-035-153; RRID: AB_2340639 |
| **Oligonucleotides and other sequence-based reagents** |  |  |
| Oligonucleotides for gRNA cloning | This study | Table EV1 |
| Repair template for ACVR1^R206H^ knock-in | This study | Table EV1 |
| Primers for ACVR1^R206H^ knock-in screening | This study | Table EV1 |
| Primers for ACVR2A cloning | This study | Table EV1 |
| Primers for ACVR2A site directed mutagenesis | This study | Table EV1 |
| Primers for ACVR1 site directed mutagenesis | This study | Table EV1 |
| Primers for qPCR | This study | Table EV1 |
| Primers for Opto-ACVR1B* cloning | This study | Table EV1 |
| **Chemicals, Enzymes and other reagents** |  |  |
| Activin A | PeproTech | Cat#120-14 |
| Activin B | PeproTech | Cat#120-15 |
| TGF-β | PeproTech | Cat#100-21C |
| BMP4 | PeproTech | Cat#120-05ET |
| BMP7 | PeproTech | Cat#120-03 |
| BMP4/7 heterodimer | R&D Systems | Cat#3727-BP/CF |
| BMP2 | R&D Systems | Cat#355-BM/CF |
| H-EGF | Shenandoah Biotech | Cat#100-26 |
| H-FGF-basic | Shenandoah Biotech | Cat#100-146 |
| H-PDGF-AA | Shenandoah Biotech | Cat#100-16 |
| H-PDGF-BB | Shenandoah Biotech | Cat#100-18 |
| Heparin Solution, 0.2% | StemCell Technologies, lnc. | Cat#15240-096 |
| B-27 Supplement Minus Vitamin A | Invitrogen | Cat#12587-010 |
| Follistatin | Sigma | Cat#F2177 |
| Atto 647N NHS ester | Sigma-Aldrich | Cat#18373 |
| CF640R, Succinimidyl Ester | Insight Biotechnology | Cat#92108 |
| rhACVR2A Fc Chimera | R&D Systems | Cat#340-RC2 |
| rhACVR2B Fc Chimera | R&D Systems | Cat#339-RB-100/CF |
| SB-431542 | Tocris | Cat#1614/1 |
| SB-505124 | Tocris | Cat#3263/10 |
| LDN-193189 | Paul Yu, Harvard Medical School, USA | N/A |
| CellBrite Green cytoplasmic membrane dye | Biotium | 30021 |
| DOPC-NiNTA | Avanti | 850375 |
| DOGS-NiNTA | Avanti | 790404 |
| NIP_1_-H12-Hylight647 | Anaspec | Custom order, AA sequence: NIP-ASTGKTASAC[HyLight647]TSGASSTGSH12 |
| **Software** |  |  |
| FIJI/ImageJ | N/A | https://imagej.net/Fiji/Downloads |
| simFCS | Laboratory for Fluorescence Dynamics | <https://www.lfd.uci.edu/globals/> |
| Metamorph | Molecular Devices |  |
| ZEN 3.0 (black edition) | Carl Zeiss | <https://www.zeiss.com/microscopy/int/products/microscope-software.html> |
| Prism 8 | GraphPad | <https://www.graphpad.com/scientific-software/prism/> |
| Matlab | MathWorks |  |
| **Other** |  |  |
| DMEM | Thermo Fisher Scientific | Cat#41966052 |
| DMEM, phenol free | Thermo Fisher Scientific | Cat#21063029 |
| OptiMEM | Thermo Fisher Scientific | Cat#31985070 |
| Neurobasal-A Medium | Invitrogen | Cat#10888-022 |
| D-MEM/F-12 (1X), liquid 1:1 | Invitrogen | Cat#11330-032 |
| HEPES Buffer Solution (1M) | Invitrogen | Cat#15630-080 |
| MEM Non-Essential Amino Acids | Invitrogen | Cat#11140-050 |
| GlutaMAX-I Supplement | Invitrogen | Cat#35050-061 |
| Antibiotic-Antimytotic | Invitrogen | Cat#15240-096 |
| Penicillin/streptomycin | Thermo Fisher Scientific | Cat#15140122 |
| Sodium Pyruvate | Thermo Fisher Scientific | Cat#11360070 |
| Poly-l-lysine solution, 0.01%, STERILE-F | Sigma-Aldrich | Cat#P4707 |
| Cultrex 3D Culture Matrix Laminin I | R&D Systems | Cat#3446-005-01 |
| Fugene HD | Promega | Cat#E2312 |
| PowerUp SYBR Green Master Mix | Thermo Fisher | A25742 |
| Quickextract DNA extraction solution | Lucigen | Cat#QE09050 |
