# Supplementary material for: Pathogenic ACVR1R206H activation by Activin A‐induced receptor clustering and autophosphorylation
Source: EMBO J. 2021 May 18;40(14):e106317. doi: 10.15252/embj.2020106317 (PMC8280795; doi:10.15252/embj.2020106317)
Supplement: Supplementary file 5 — Movie EV1 [file EMBJ-40-e106317-s011.zip › EMBOJ-2020-106317R_MovieEV1/Legend to Movie EV1.docx]

**Movie EV1.**

Automated time-lapse TIRF imaging of (NIP)_1_-H12-Hylight647 containing bilayer for 20 seconds (201 frames, 100 ms exposure).
