# Supplementary material for: Pathogenic ACVR1R206H activation by Activin A‐induced receptor clustering and autophosphorylation
Source: EMBO J. 2021 May 18;40(14):e106317. doi: 10.15252/embj.2020106317 (PMC8280795; doi:10.15252/embj.2020106317)
Supplement: Supplementary file 6 — Movie EV2 [file EMBJ-40-e106317-s013.zip › EMBOJ-2020-106317R_MovieEV2/Legend to Movie EV2.docx]

**Movie EV2.**

Automated time-lapse TIRF imaging of HOM1 cells plated on (NIP)_1_-H12-Hylight647 containing bilayer. Cells were imaged every 40 seconds, during a 20 min time period (41 frames, 100 ms exposure). This movie is from another biological replicate than the one shown in Fig. 7A.
