# Supplementary material for: Pathogenic ACVR1R206H activation by Activin A‐induced receptor clustering and autophosphorylation
Source: EMBO J. 2021 May 18;40(14):e106317. doi: 10.15252/embj.2020106317 (PMC8280795; doi:10.15252/embj.2020106317)
Supplement: Supplementary file 7 — Movie EV3 [file EMBJ-40-e106317-s001.zip › EMBOJ-2020-106317R_MovieEV3/Legend to Movie EV3.docx]

**Movie EV3.**

Automated time-lapse TIRF imaging of His-Activin A Atto647N containing lipid bilayer for 20 seconds (201 frames, 100 ms exposure). Three time-points from this movie are presented in Fig. 7C.
