# Supplementary material for: Pathogenic ACVR1R206H activation by Activin A‐induced receptor clustering and autophosphorylation
Source: EMBO J. 2021 May 18;40(14):e106317. doi: 10.15252/embj.2020106317 (PMC8280795; doi:10.15252/embj.2020106317)
Supplement: Supplementary file 8 — Movie EV4 [file EMBJ-40-e106317-s017.zip › EMBOJ-2020-106317R_MovieEV4/Legend to Movie EV4.docx]

**Movie EV4.**

Automated time-lapse TIRF imaging of HOM1 cells plated on His-Activin A Atto647N containing lipid bilayer. Cells were imaged as in Movie EV1. This movie is from another biological replicate than the one shown in Fig. 7A.
