# Supplementary material for: Pathogenic ACVR1R206H activation by Activin A‐induced receptor clustering and autophosphorylation
Source: EMBO J. 2021 May 18;40(14):e106317. doi: 10.15252/embj.2020106317 (PMC8280795; doi:10.15252/embj.2020106317)
Supplement: Supplementary file 10 — Movie EV6 [file EMBJ-40-e106317-s003.zip › EMBOJ-2020-106317R_MovieEV6/Legend to Movie EV6.docx]

**Movie EV6.**

Automated time-lapse TIRF imaging of His-Activin A CF640R containing lipid bilayer every 30 seconds for 7.5 min (15 frames, 30 seconds between frames, 100 ms exposure). Three time-points from this movie are presented in Fig. EV5A.
