# Supplementary material for: Pathogenic ACVR1R206H activation by Activin A‐induced receptor clustering and autophosphorylation
Source: EMBO J. 2021 May 18;40(14):e106317. doi: 10.15252/embj.2020106317 (PMC8280795; doi:10.15252/embj.2020106317)
Supplement: Supplementary file 11 — Movie EV7 [file EMBJ-40-e106317-s014.zip › EMBOJ-2020-106317R_MovieEV7/Legend to Movie EV7.docx]

**Movie EV7.**

Automated time-lapse TIRF imaging of HOM1 cells plated on His-Activin A CF640R containing lipid bilayer. Cells were imaged as in Movie EV6. Three time-points from this movie are presented in Fig. EV5B.
