# Supplementary figures and images for: Pathogenic ACVR1R206H activation by Activin A‐induced receptor clustering and autophosphorylation
Source: EMBO J. 2021 May 18;40(14):e106317. doi: 10.15252/embj.2020106317 (PMC8280795; doi:10.15252/embj.2020106317)

A

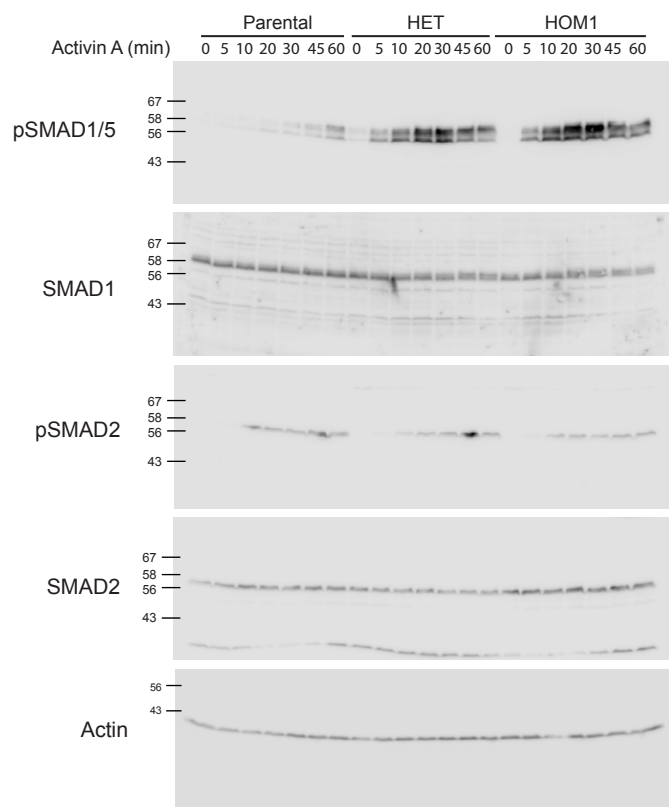

B

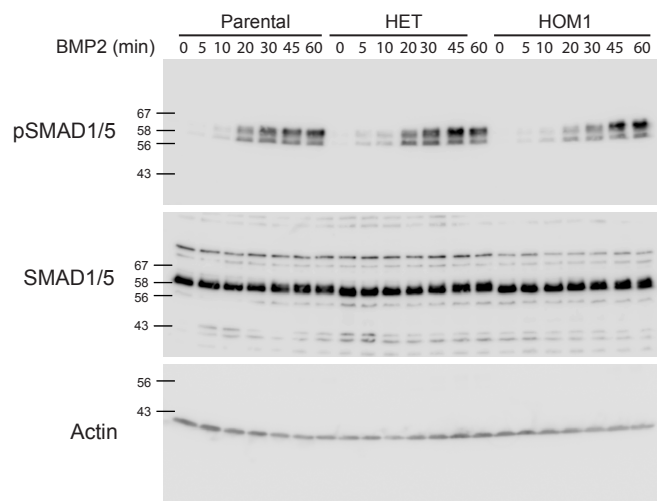

Supplement: Supplementary file 12 — Source Data for Expanded View/Appendix [file EMBJ-40-e106317-s004.zip › Source data for Expanded Figures and Appendix/EMBOJ-2020-106317R_SourceDataWBForAppendixFigure S1.pdf]

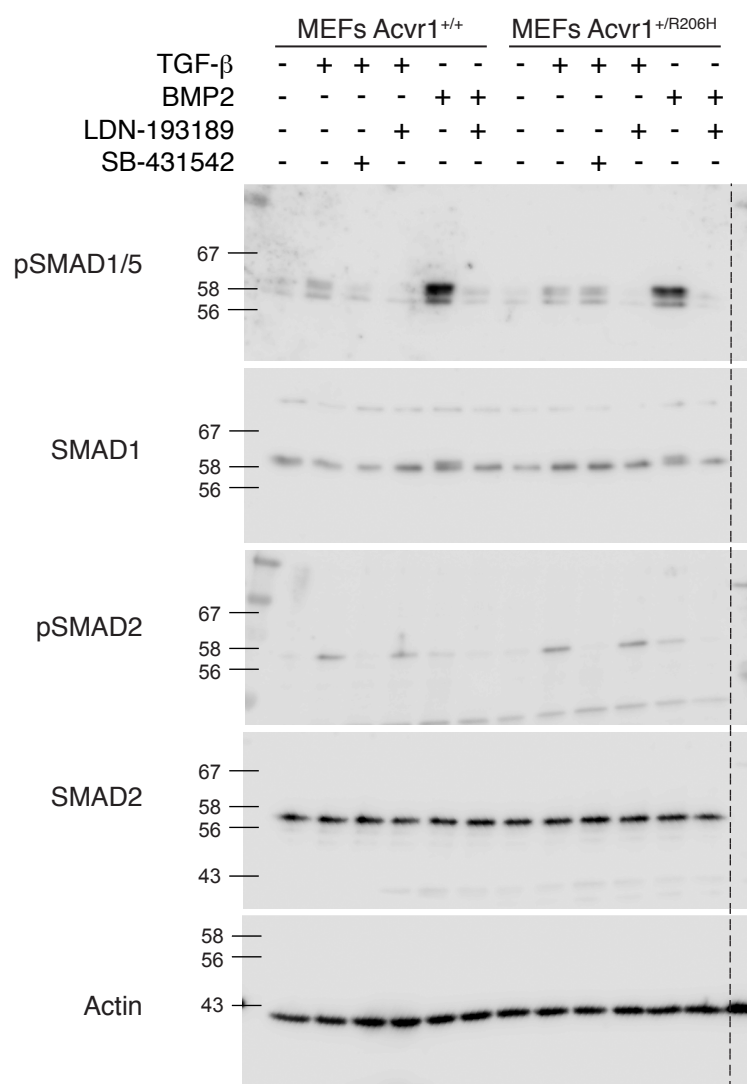

Supplement: Supplementary file 12 — Source Data for Expanded View/Appendix [file EMBJ-40-e106317-s004.zip › Source data for Expanded Figures and Appendix/EMBOJ-2020-106317R_SourceDataWBForAppendixFigureS2.pdf]

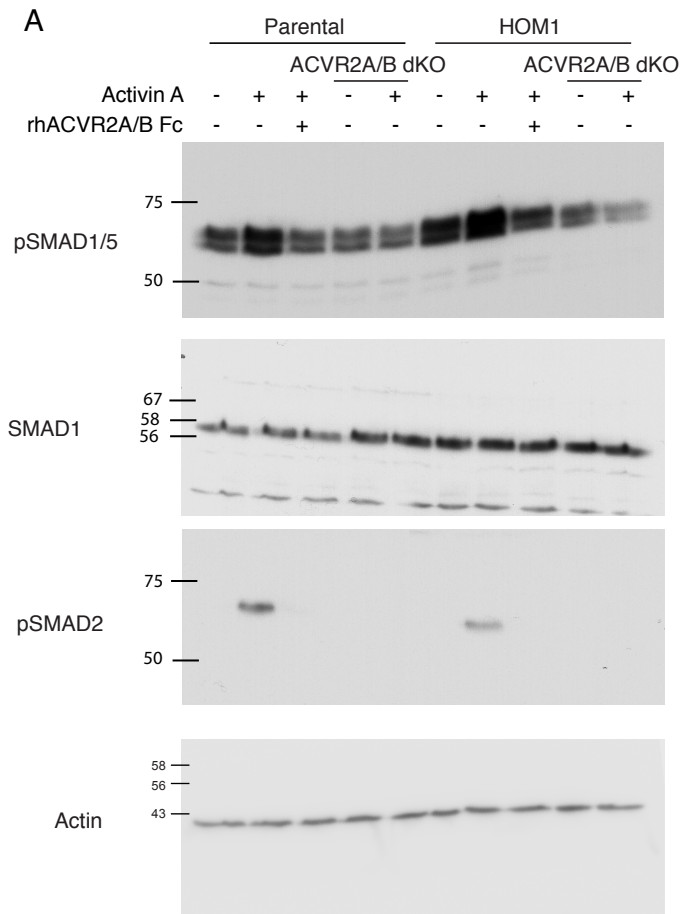

Figure EV4

Supplement: Supplementary file 12 — Source Data for Expanded View/Appendix [file EMBJ-40-e106317-s004.zip › Source data for Expanded Figures and Appendix/EMBOJ-2020-106317R_SourceDataWBForFigureEV4.pdf]

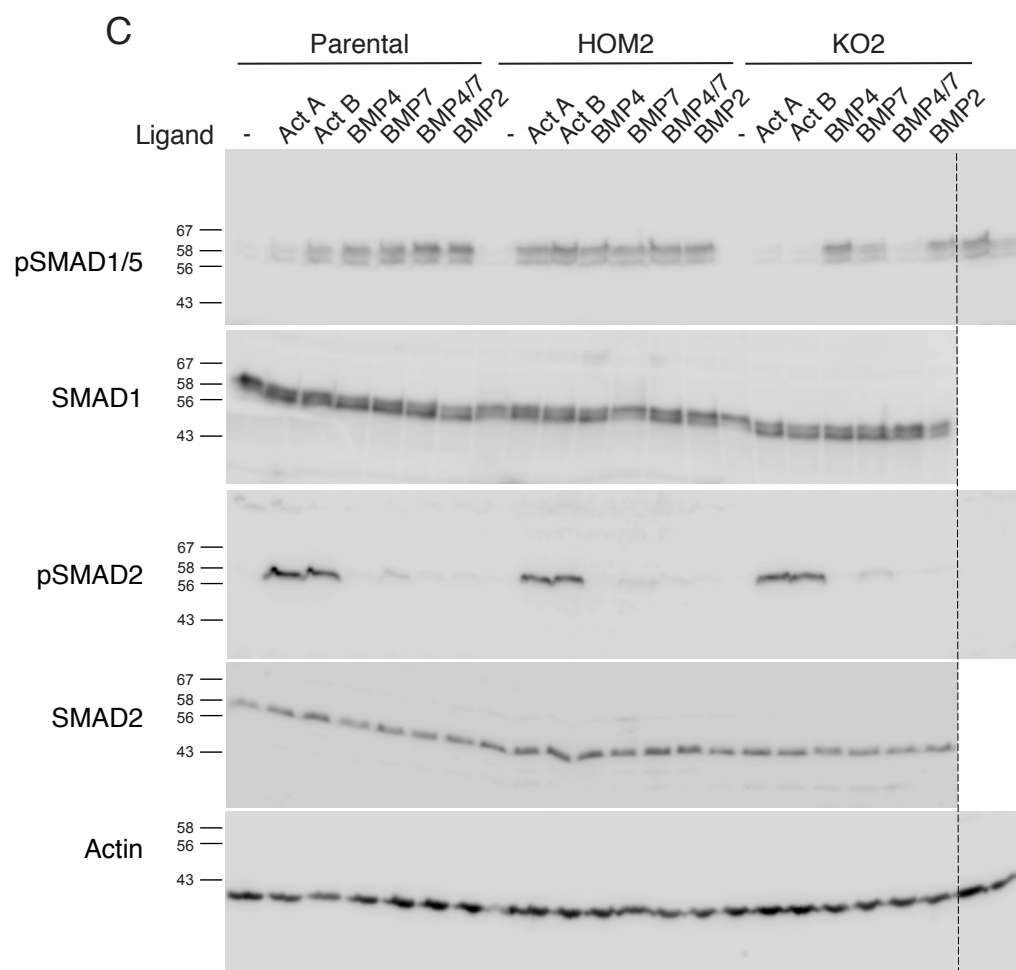

Fig. EV1

Supplement: Supplementary file 12 — Source Data for Expanded View/Appendix [file EMBJ-40-e106317-s004.zip › Source data for Expanded Figures and Appendix/EMBOJ-2020-106317R_SourceDataWBForFigureEV1.pdf]

A

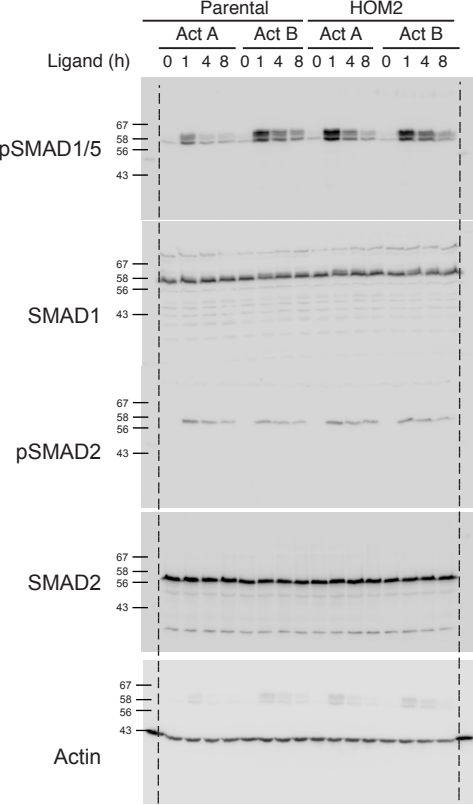

C

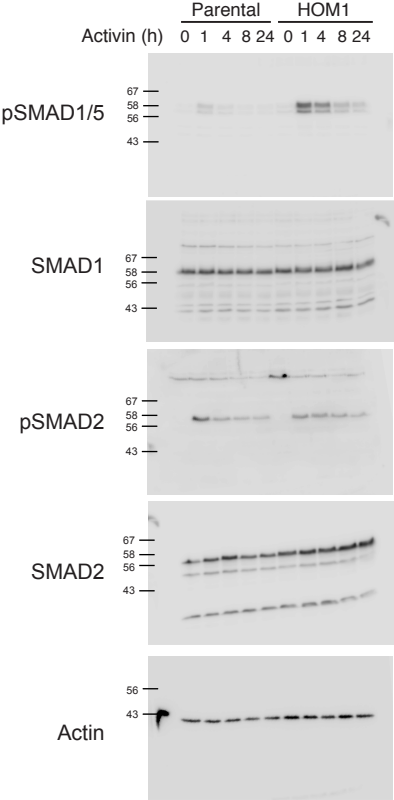

D

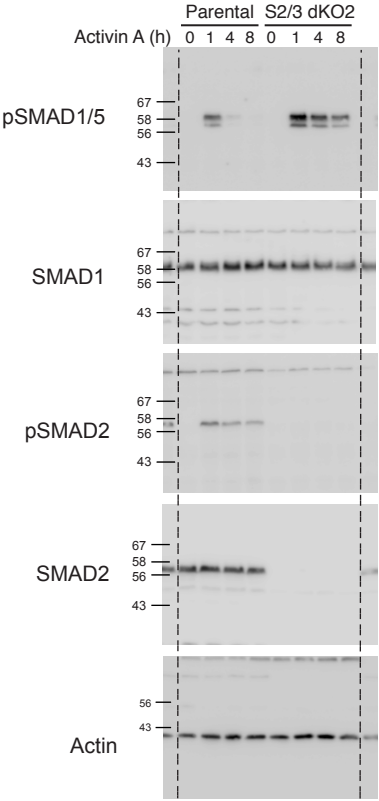

F

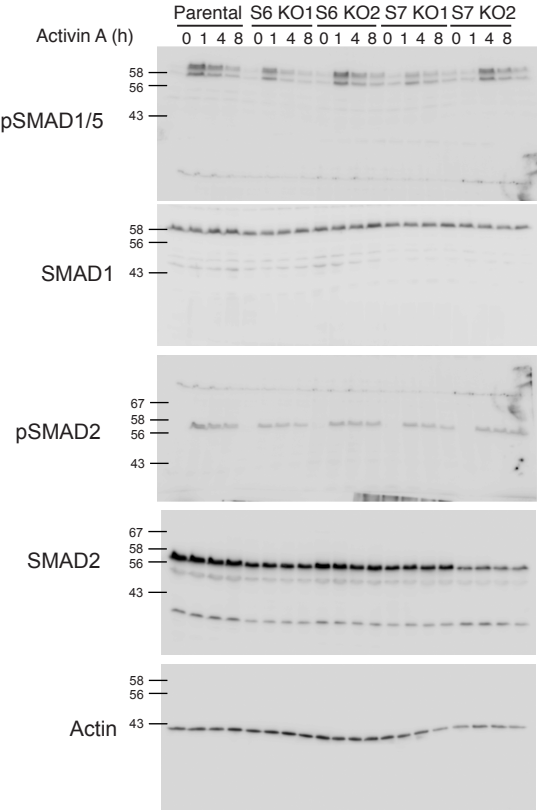

Figure EV2

Supplement: Supplementary file 12 — Source Data for Expanded View/Appendix [file EMBJ-40-e106317-s004.zip › Source data for Expanded Figures and Appendix/EMBOJ-2020-106317R_SourceDataWBForFigureEV2.pdf]

A

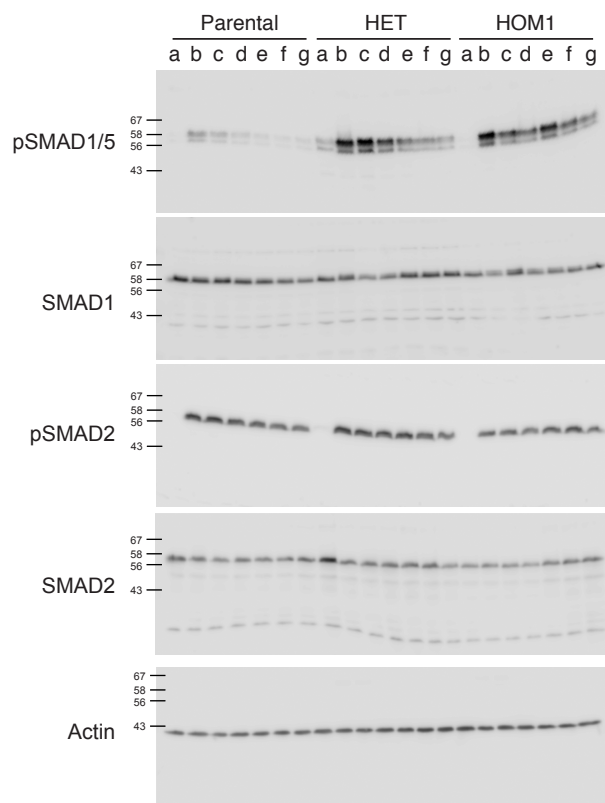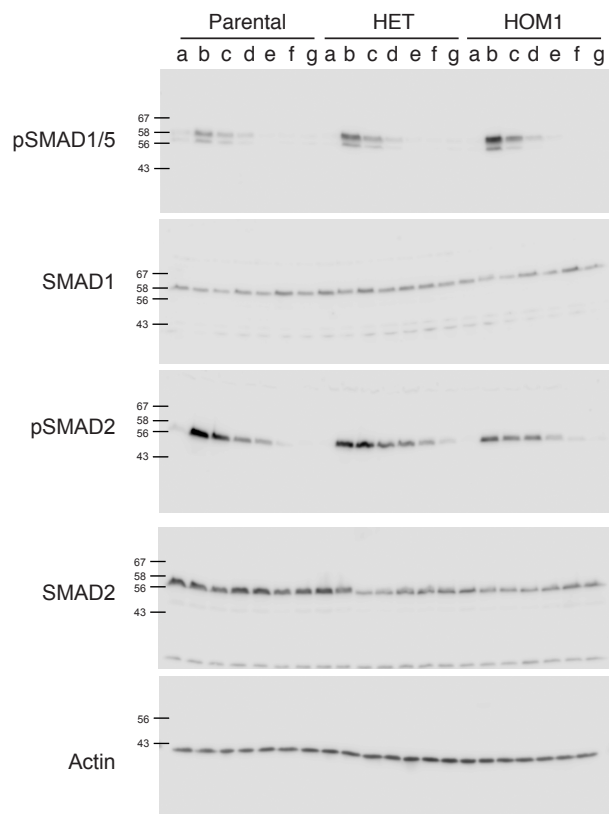

B

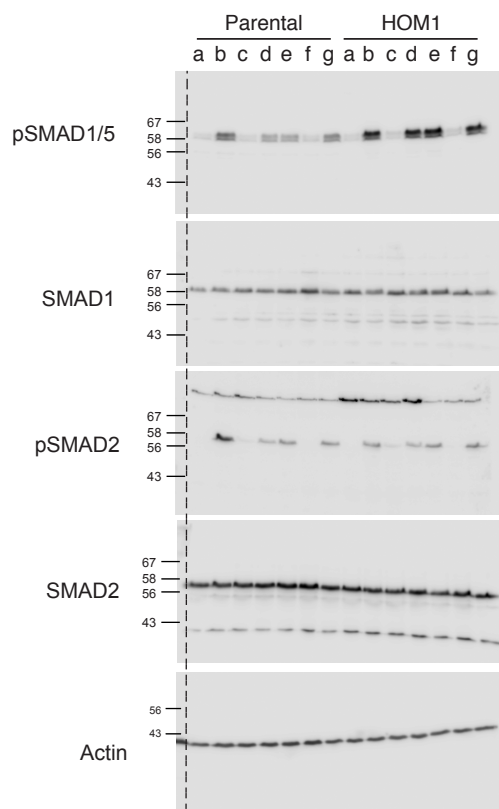

C

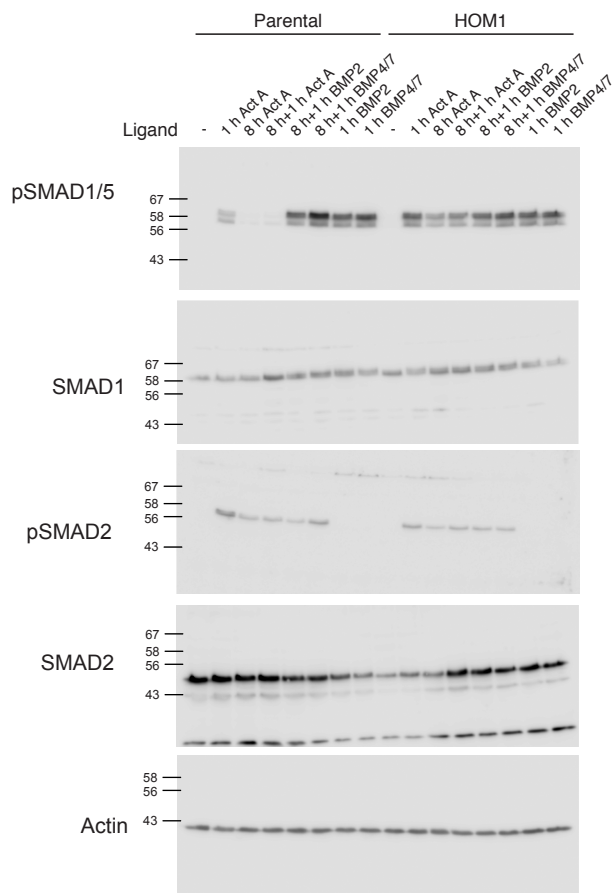

Figure EV3

Supplement: Supplementary file 12 — Source Data for Expanded View/Appendix [file EMBJ-40-e106317-s004.zip › Source data for Expanded Figures and Appendix/EMBOJ-2020-106317R_SourceDataWBForFigureEV3.pdf]

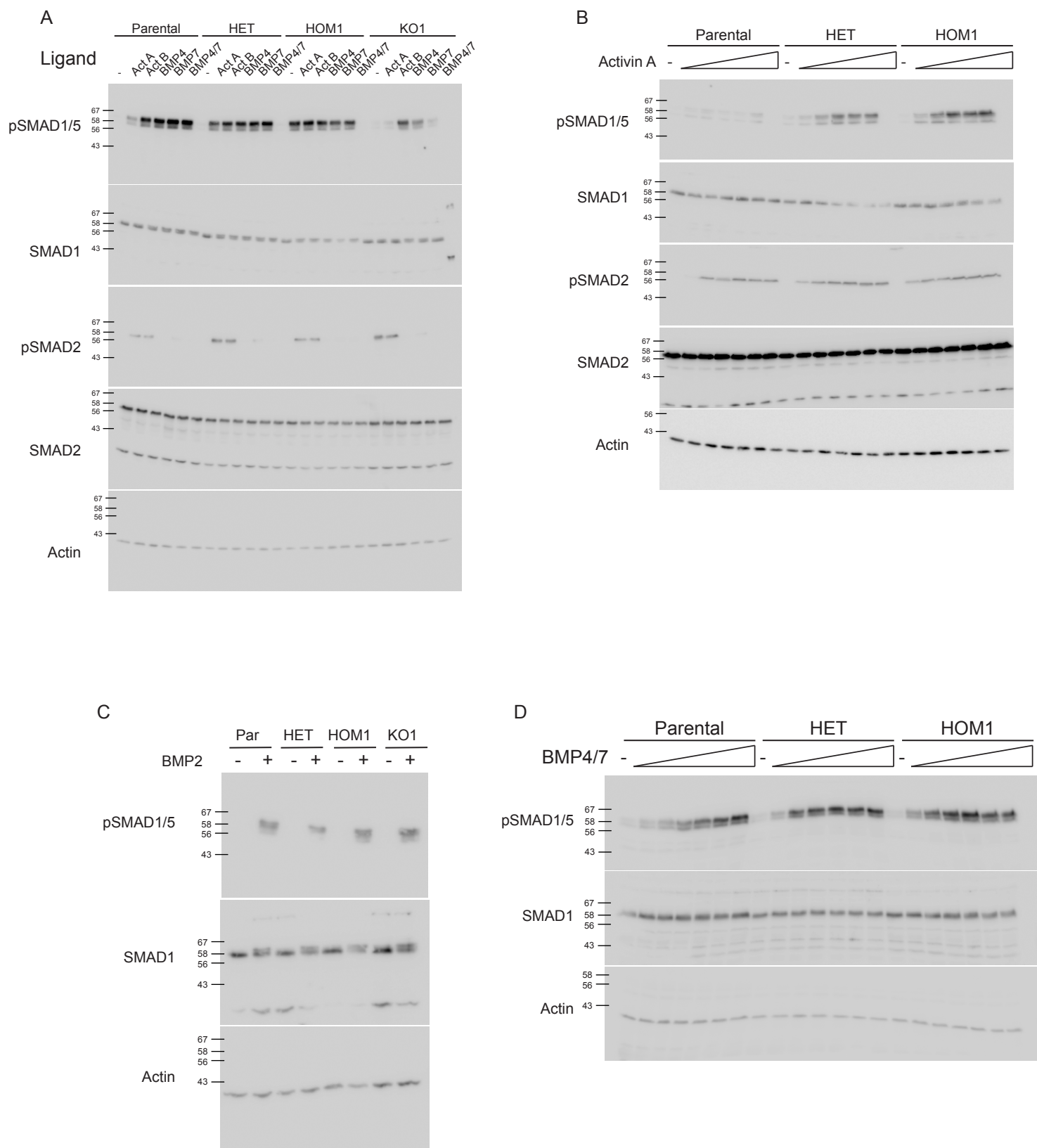

Figure 1

Supplement: Supplementary file 14 — Source Data for Figure 1 [file EMBJ-40-e106317-s007.pdf]

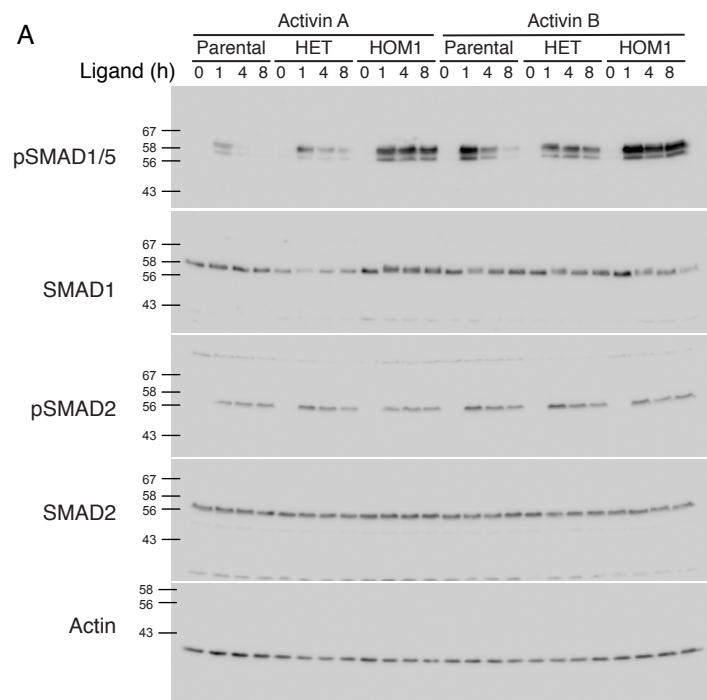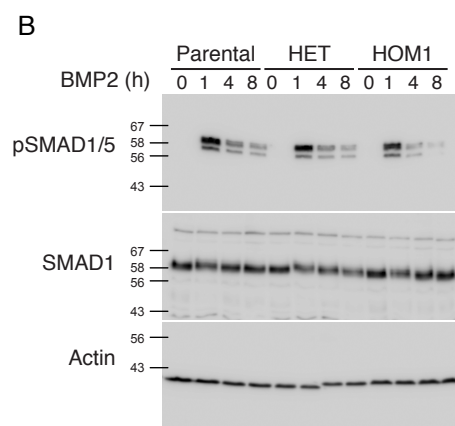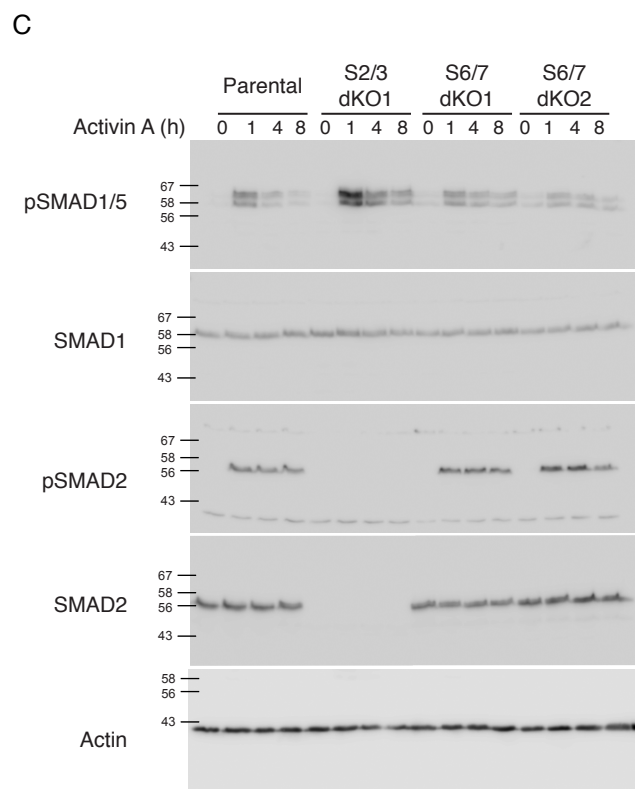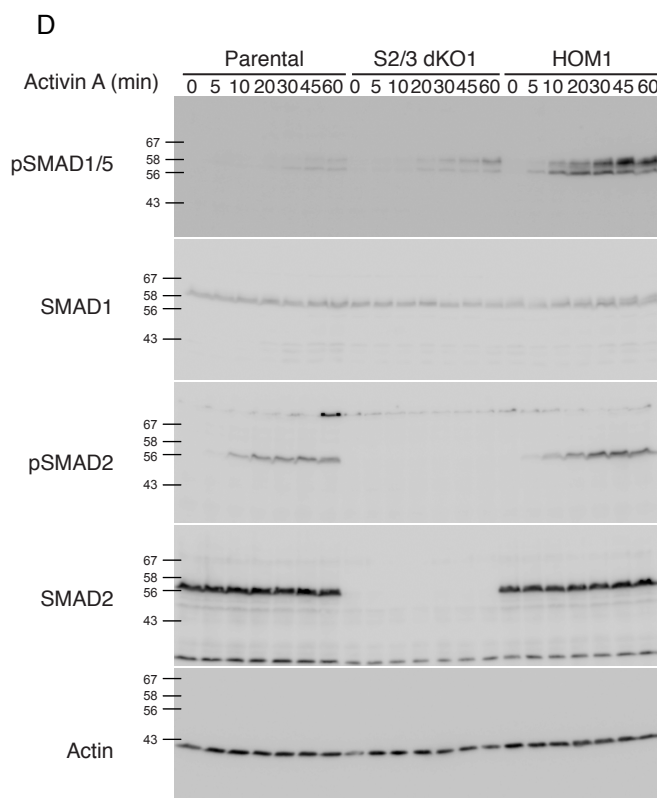

Figure 2

Supplement: Supplementary file 15 — Source Data for Figure 2 [file EMBJ-40-e106317-s008.zip › embj2020106317-sup-0013-SDataFig2.pdf]

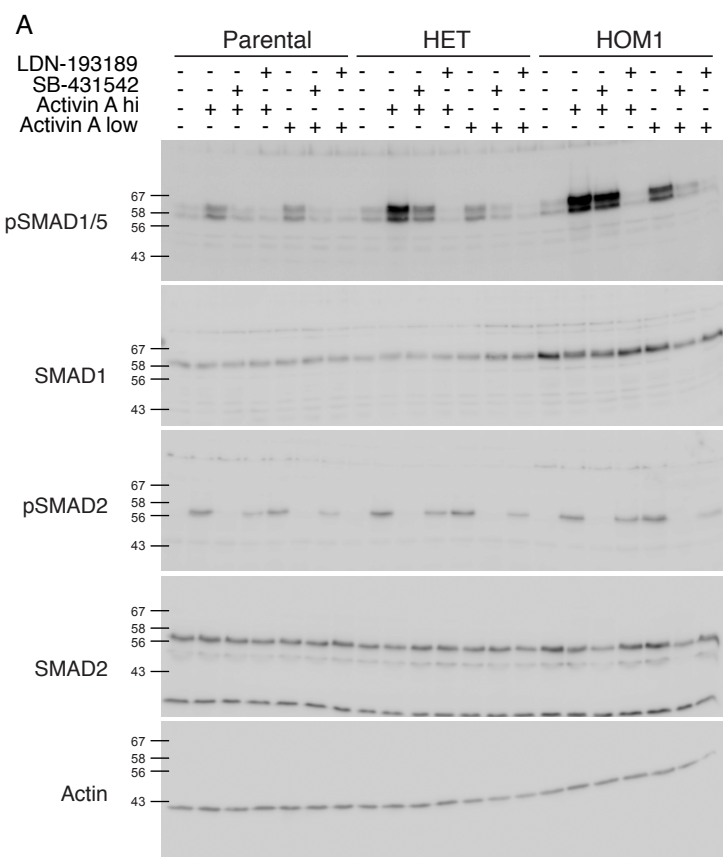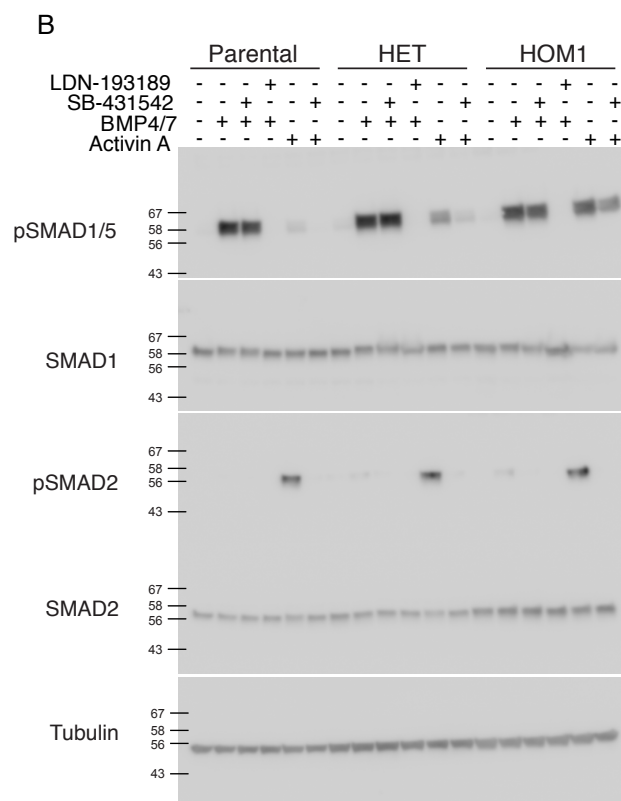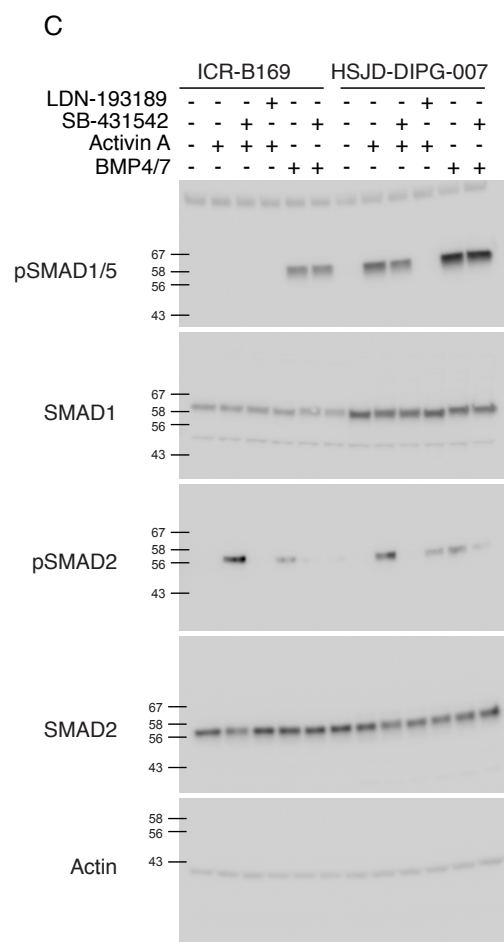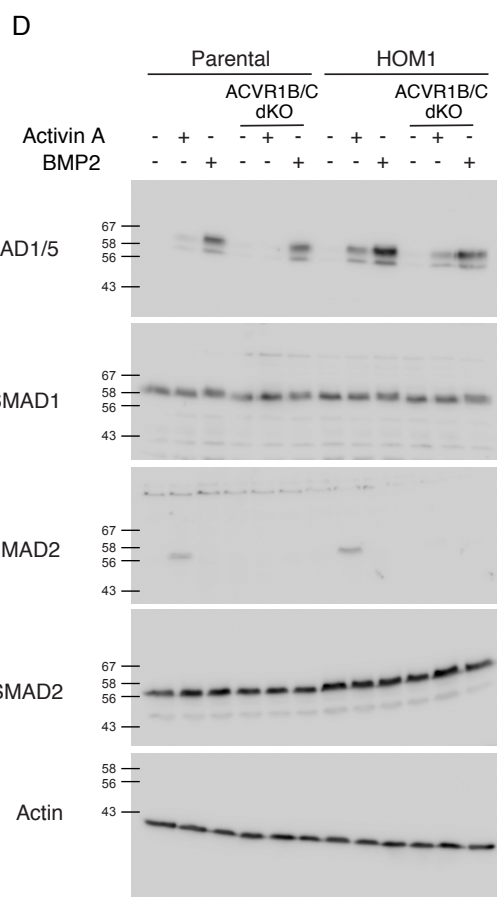

Figure 3

Supplement: Supplementary file 16 — Source Data for Figure 3 [file EMBJ-40-e106317-s018.zip › embj2020106317-sup-0015-SDataFig3.pdf]

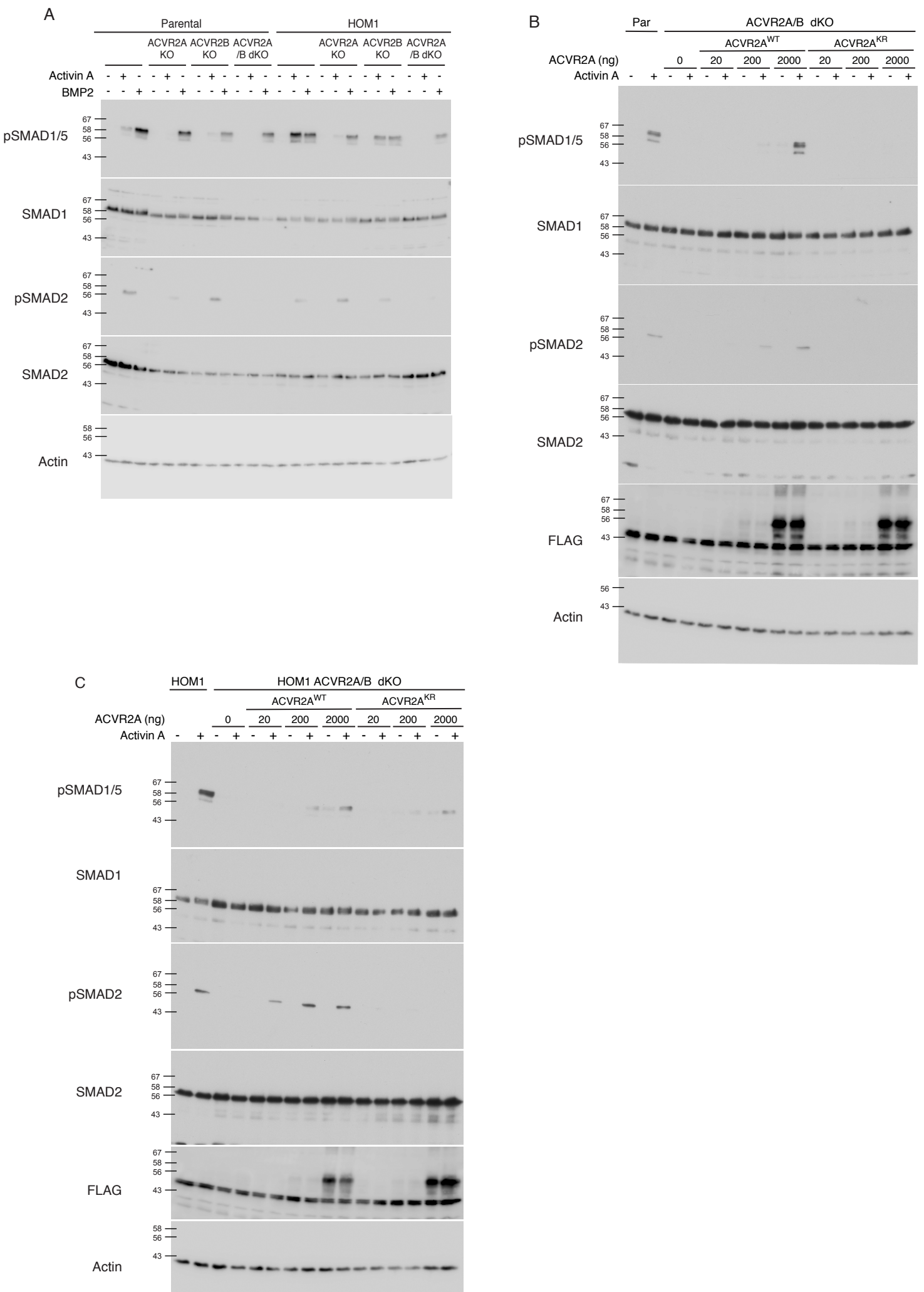

Figure 4

Supplement: Supplementary file 17 — Source Data for Figure 4 [file EMBJ-40-e106317-s020.pdf]

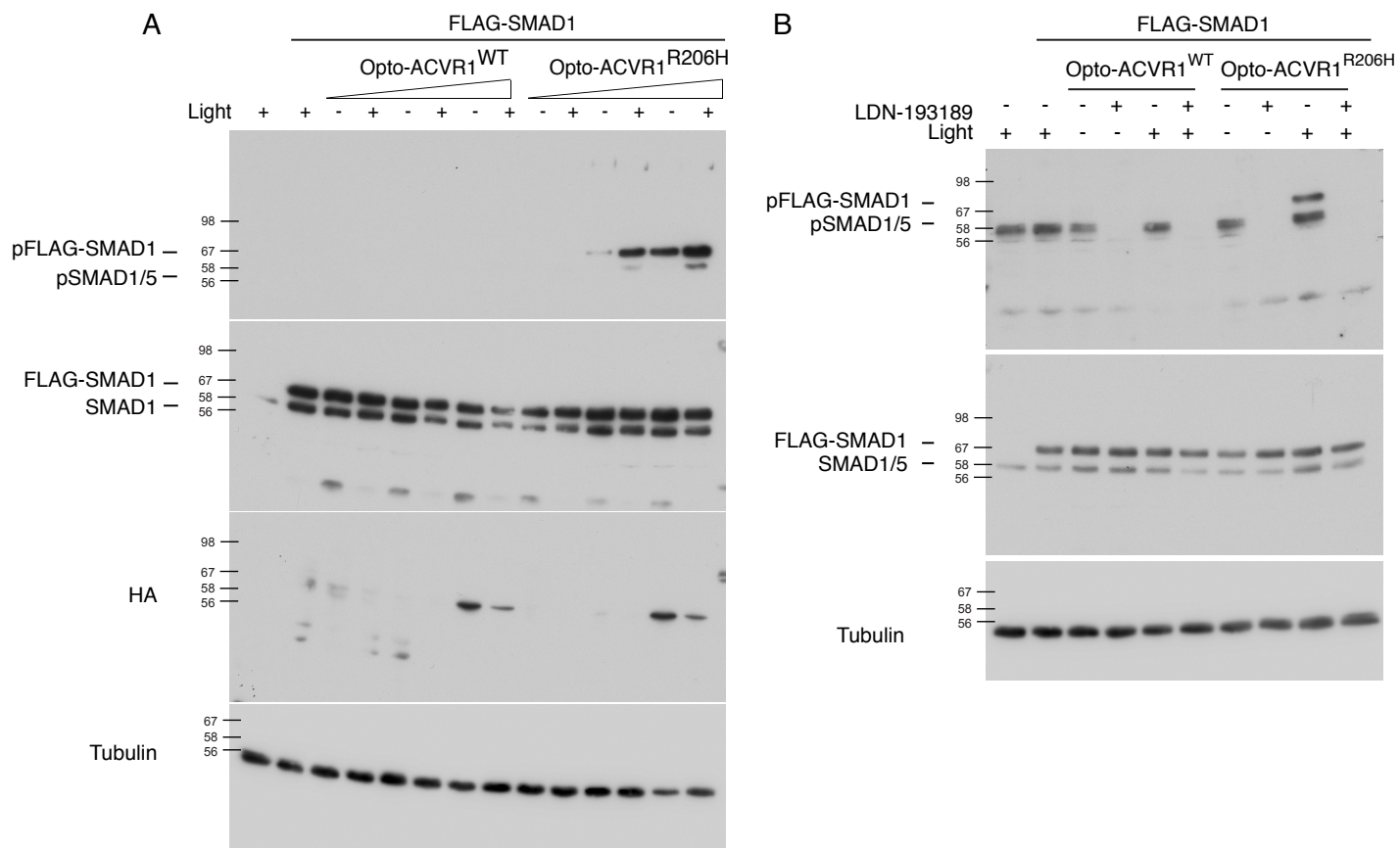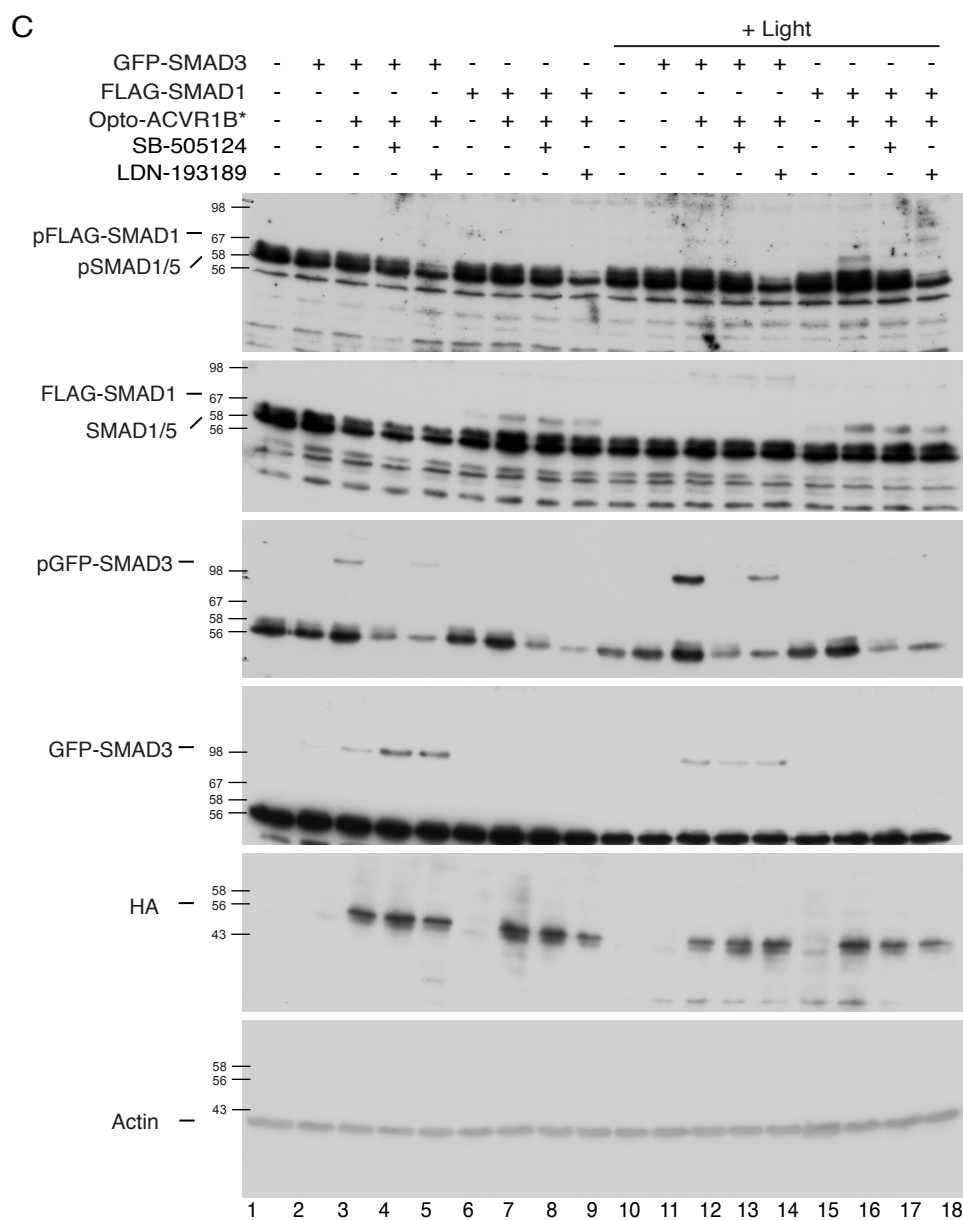

Figure 5

Supplement: Supplementary file 18 — Source Data for Figure 5 [file EMBJ-40-e106317-s012.pdf]
